# UAV multi-source data fusion with super-resolution for accurate soybean leaf area index estimation

**DOI:** 10.3389/fpls.2025.1700660

**Published:** 2025-11-20

**Authors:** Zhenqing Zhao, Huabo Yao, Depeng Zeng, Zhenfeng Jiang, Xihai Zhang

**Affiliations:** 1 College of Electrical Engineering and Information, Northeast Agricultural University, Harbin, China; 2 National Key Laboratory of Smart Farm Technologies and Systems, Harbin, China; 3 College of Agriculture, Northeast Agricultural University, Harbin, China

**Keywords:** UAV remote sensing, machine learning, super resolution, leaf area index, multi-source data fusion

## Abstract

**Introduction:**

The Leaf Area Index (LAI) is a critical biophysical parameter for assessing crop canopy structure and health. Unmanned Aerial Vehicles (UAVs) equipped with multispectral sensors offer a high-throughput solution for LAI estimation, but flight altitude compromises between efficiency and image resolution, ultimately impacting accuracy. This study investigates the integration of super-resolution (SR) image reconstruction with multi-sensor data to enhance LAI estimation for soybeans across varying UAV flight altitudes.

**Methods:**

RGB and multispectral images were captured at four flight altitudes: 15 m, 30 m, 45 m, and 60 m. The acquired images were processed using several SR algorithms (SwinIR, Real-ESRGAN, SRCNN, and EDSR). Texture features were extracted from the RGB images, and LAI estimation models were developed using the XGBoost algorithm, testing data fusion strategies that included RGB-only, multispectral-only, and a combined RGB-multispectral approach.

**Results:**

(1) SR performance declined with increasing altitude, with SwinIR achieving superior image reconstruction quality (PSNR and SSIM) over other methods. (2) Texture features from RGB images showed strong sensitivity to LAI. The XGBoost model leveraging fused RGB and multispectral data achieved the highest accuracy (relative error: 4.16%), outperforming models using only RGB (5.25%) or only multispectral data (9.17%). (3) The application of SR techniques significantly improved model accuracy at 30 m and 45 m altitudes. At 30 m, models incorporating Real-ESRGAN and SwinIR achieved an average R^2^ of 0.86, while at 45 m, these methods yielded models with an average R^2^ of 0.77.

**Discussion:**

The results demonstrate that the fusion of SR-reconstructed imagery with multi-sensor data can effectively mitigate the negative impact of higher flight altitudes on LAI estimation accuracy. This approach provides a robust and efficient framework for UAV-based crop monitoring, enhancing data-driven decision-making in precision agriculture.

## 1 Introduction

Soybean is a globally important food and oil crop that contributes significantly to food security, serving as a major source of high-quality vegetable protein and essential fatty acids for both human and animal nutrition (Hartman et al., 2011). As both a source of protein and oil, soybeans play a crucial role in enhancing global food security and sustainable development (Chen et al., 2022). The Leaf Area Index (LAI), defined as the area of leaves per unit surface area of a field crop with flat leaves (Watson, 1947), measures the density and extent of vegetation leaf cover (Su et al., 2019). LAI is a critical structural variable in vegetation, indicative of crop growth and health, and is thus employed as an input variable in models that predict crop growth and yield (Fang et al., 2019).

The measurement of crop LAI involves a range of techniques, categorized into direct and indirect methods (Strachan et al., 2005). The direct method typically requires personnel to collect leaf samples from various locations and measure the length, maximum width, and number of leaves. These data are then used to calculate LAI (Shi et al., 2022). Indirect methods, on the other hand, include close-range detection techniques such as the use of handheld measuring devices and fisheye lens photography (Confalonieri et al., 2013), among others. Both methods, however, have limitations regarding sampling range and efficiency.

The adoption of remote sensing technology presents a more effective approach to deriving crop LAI from spectral data (Sadeh et al., 2021; Dhakar et al., 2021). Recent advances have demonstrated that UAV-based multispectral imaging can substantially improve the accuracy of crop LAI and chlorophyll estimation, benefiting from its high spatial resolution and spectral sensitivity (Parida et al., 2024). This technology significantly mitigates the constraints of traditional measurement methods, allowing researchers to economically and swiftly gather extensive crop canopy data (Furbank and Tester, 2011). Nonetheless, the temporal and spatial resolutions of most satellite data do not meet the rigorous demands of precision agriculture. In this context, Unmanned Aerial Vehicle (UAV) technology plays a pivotal role by facilitating high-throughput phenotypic analysis (Chapman et al., 2018). The integration of UAV-based LiDAR and multispectral imaging has proven effective for high-throughput phenotyping of dry bean, enabling accurate, non-destructive estimation of plant height, lodging, and seed yield, highlighting the potential of UAV-based phenotyping in precision agriculture and crop breeding programs (Panigrahi et al., 2025).Offering lower altitudes and capturing data with higher spatial resolution, UAVs provide more detailed information compared to satellite data (Araus and Cairns, 2014). Combining UAV RGB and multispectral indices enables accurate field-scale monitoring of nitrogen status and yield prediction (Chatraei Azizabadi et al., 2025).In addition, multi-source UAV data fusion frameworks have been developed for real-time LAI estimation, highlighting the importance of integrating structural and spectral information (Du et al., 2024).

The flight efficiency of UAVs varies with flying altitude. In summary, the time required for large-scale data collection presents significant challenges to precision agriculture. To reduce collection costs, an increase in flying altitude is necessary; however, this compromises image quality and decreases image resolution. Previous studies have typically adjusted flight altitudes to balance the relationship between flight duration and data accuracy (Jay et al., 2019). However, high-resolution sensors are often costly and limited in coverage, while low-altitude flights are time-consuming and less practical for large-scale monitoring. Therefore, a cost-effective solution is needed to enhance image resolution without additional flight or hardware costs. To address the issue of low resolution in high-altitude imagery, employing Image Super-Resolution (SR) algorithms is considered an appropriate method (Jonak et al., 2024). Image SR involves reconstructing low-resolution images (LR) into high-resolution images (HR) using computer vision algorithms. In remote sensing, SR technology enhances image detail, resolution, and quality through AI models, proving to be of extensive and practical value in fields such as land cover classification, crop monitoring, and object detection. In particular, SR not only improves visual quality but also enhances the extraction of fine textural and structural features from UAV imagery, thereby potentially improving model robustness and predictive accuracy for biophysical variables such as LAI. Nevertheless, few studies have combined UAV-based SR imagery with multispectral data for quantitative crop trait estimation such as LAI, especially for soybean canopies.

This study introduces a novel method that utilizes UAVs and SR techniques to estimate the LAI. By enhancing the resolution of the collected RGB images by a factor of four, the clarity of these images is improved, thereby minimizing the impact of image resolution on feature detection and enhancing the precision of the model. Additionally, this research develops an integrated learning regression model that combines light and multispectral (MS) information, increasing both the model’s precision and computational efficiency. This approach aims to address the current gap in linking SR-enhanced UAV data with multispectral information for high-accuracy soybean LAI estimation. The goals of this study are to: (1) enhance the resolution of UAV imagery using SR techniques and use these enhanced images to estimate the LAI of soybeans, selecting an appropriate estimation model; (2) determine the combination that yields the highest precision in a multivariate remote sensing imagery dataset (RGB+MS, MS, RGB); (3) assess the key features associated with the LAI.

## 2 Material and methods

### 2.1 Study site

**Figure 1:**
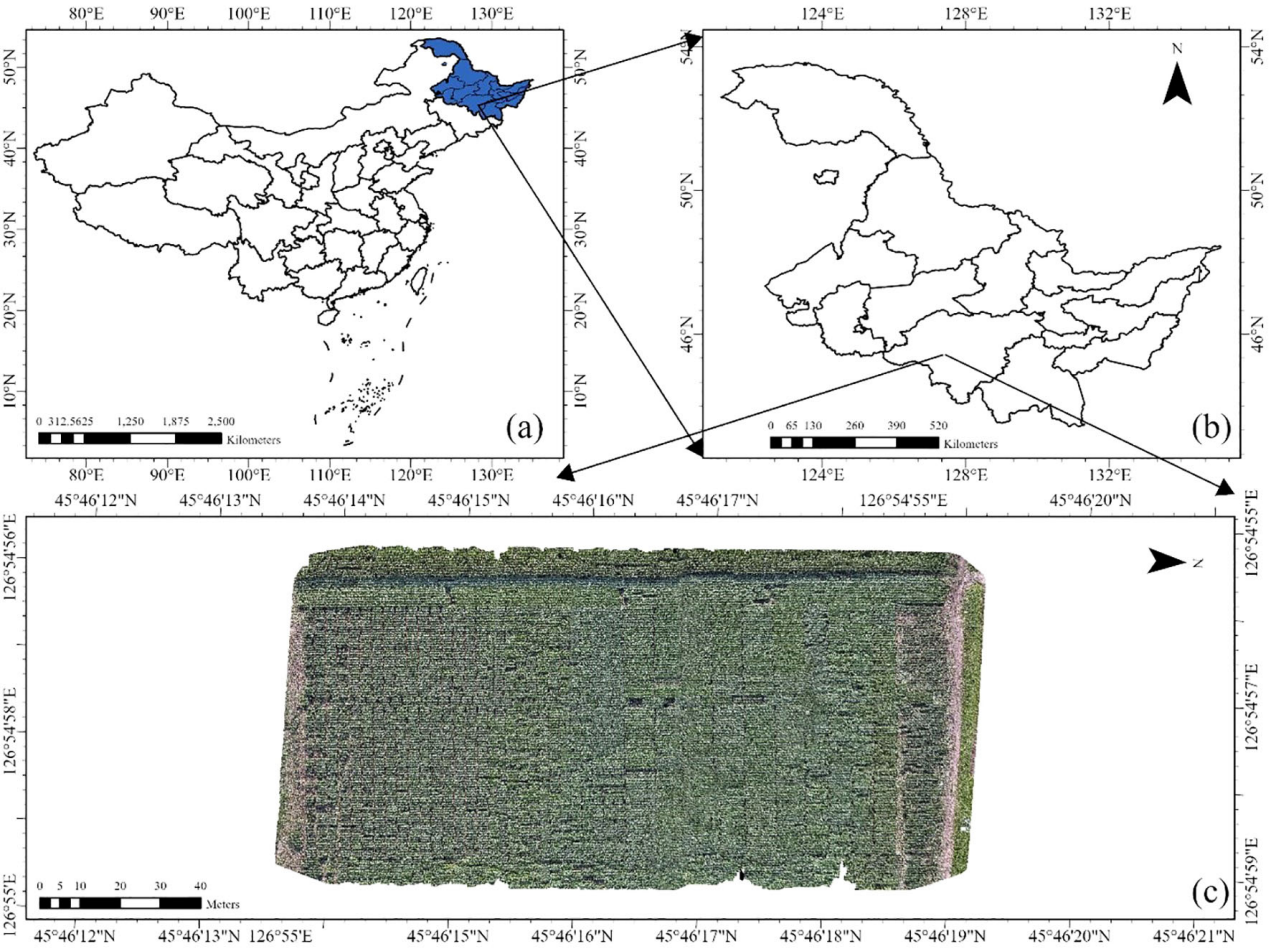
Location of the study area and experimental site design at the Xiangyang site of Northeast Agricultural University in Harbin, Heilongjiang Province, China. **(a)** Geographic location of Heilongjiang Province (highlighted in blue) within China, **(b)** Map of Heilongjiang Province showing the specific location of the study area, **(c)** Layout of the experimental areas and treatments at the Xiangyang site.

As shown in **Figure 1**, the study was conducted in 2024 at the Xiangyang Farm, located in Harbin City, Heilongjiang Province, China, within the Northeast Plain—one of the world’s three largest black soil regions. This area is renowned for its fertile soil, which is particularly well-suited for soybean cultivation. Heilongjiang Province, where the site is located, is responsible for producing over 50% of China’s soybean output (National Bureau of Statistics of China, 2022). The geographic coordinates of the site are 45°45’N latitude and 126°54’E longitude, with an average elevation of approximately 150 m. The region experiences a temperate monsoon climate, characterized by an average annual precipitation of 552.9 mm and an average annual temperature of 3.8°C. The majority of the rainfall, over 70%, occurs between May and August (Wikipedia contributors, 2025). In May 2024, 100 soybean varieties, selected for their suitability in Northeast China and varying in yield and growth periods, were planted. These varieties were also used for model validation to assess the robustness of the estimation results across different genotypes. The names of all varieties are listed in **Appendix Table A2**.The management of pesticides and fertilizers adhered to local practices.

### 2.2 LAI estimation framework

**Figure 2:**
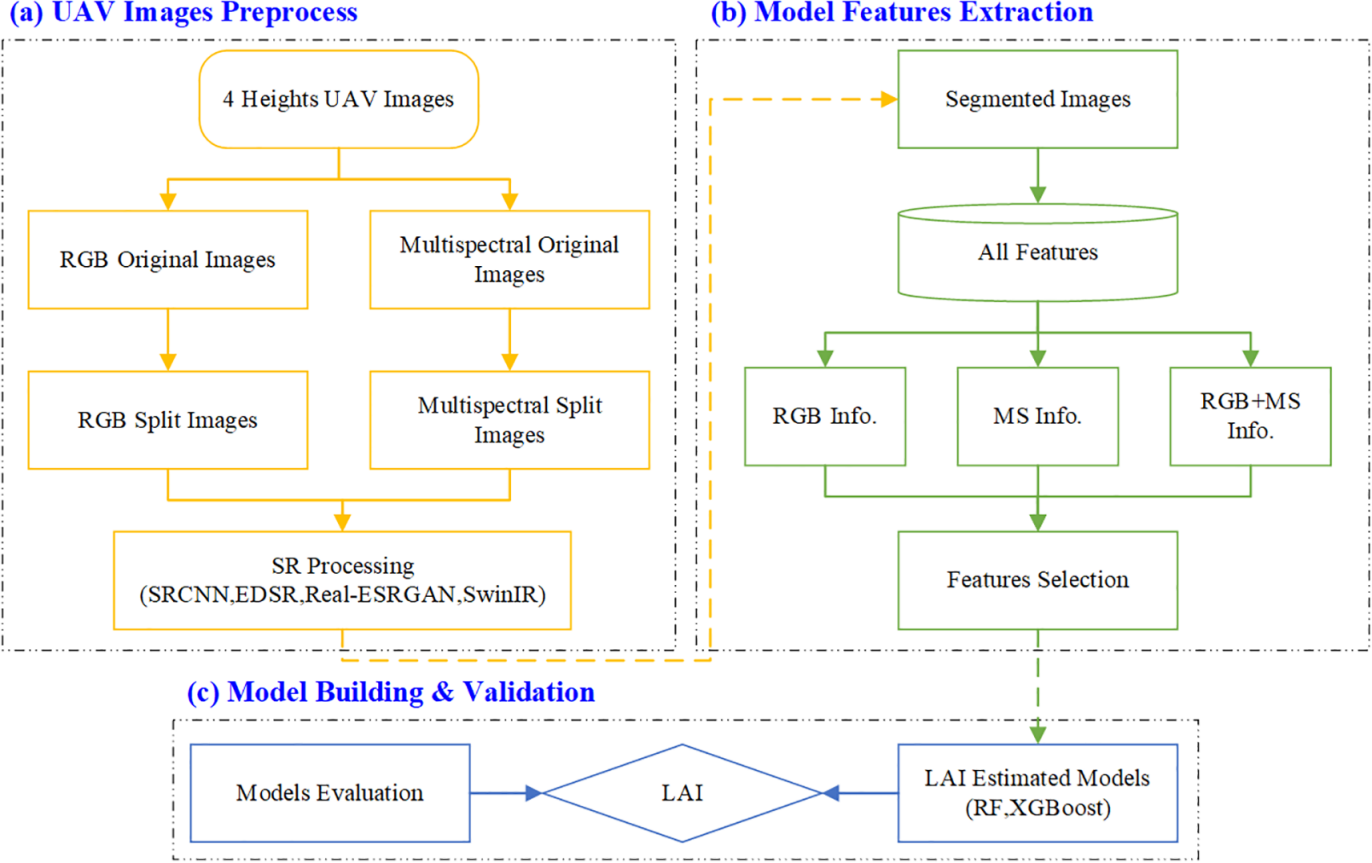
Workflow diagram of the proposed methodology. **(a)** UAV Images Preprocess, **(b)** Model Features Extraction, **(c)** Model Building & Validation.

This study developed a framework for estimating the LAI using UAV-based imagery. As depicted in **Figure 2**, the framework involves a comprehensive workflow that includes image preprocessing using SR techniques, integrating three distinct types of input features, and employing a regression model for LAI estimation. Initially, RGB images at four different altitudes were captured using a UAV. These images underwent preprocessing, which included stitching and segmentation. The segmented images were then enhanced in resolution through four SR methods. The next phase involved extracting features from these enhanced images and determining three different combinations of input features for LAI estimation.

### 2.3 Field data collection

**Figure 3:**
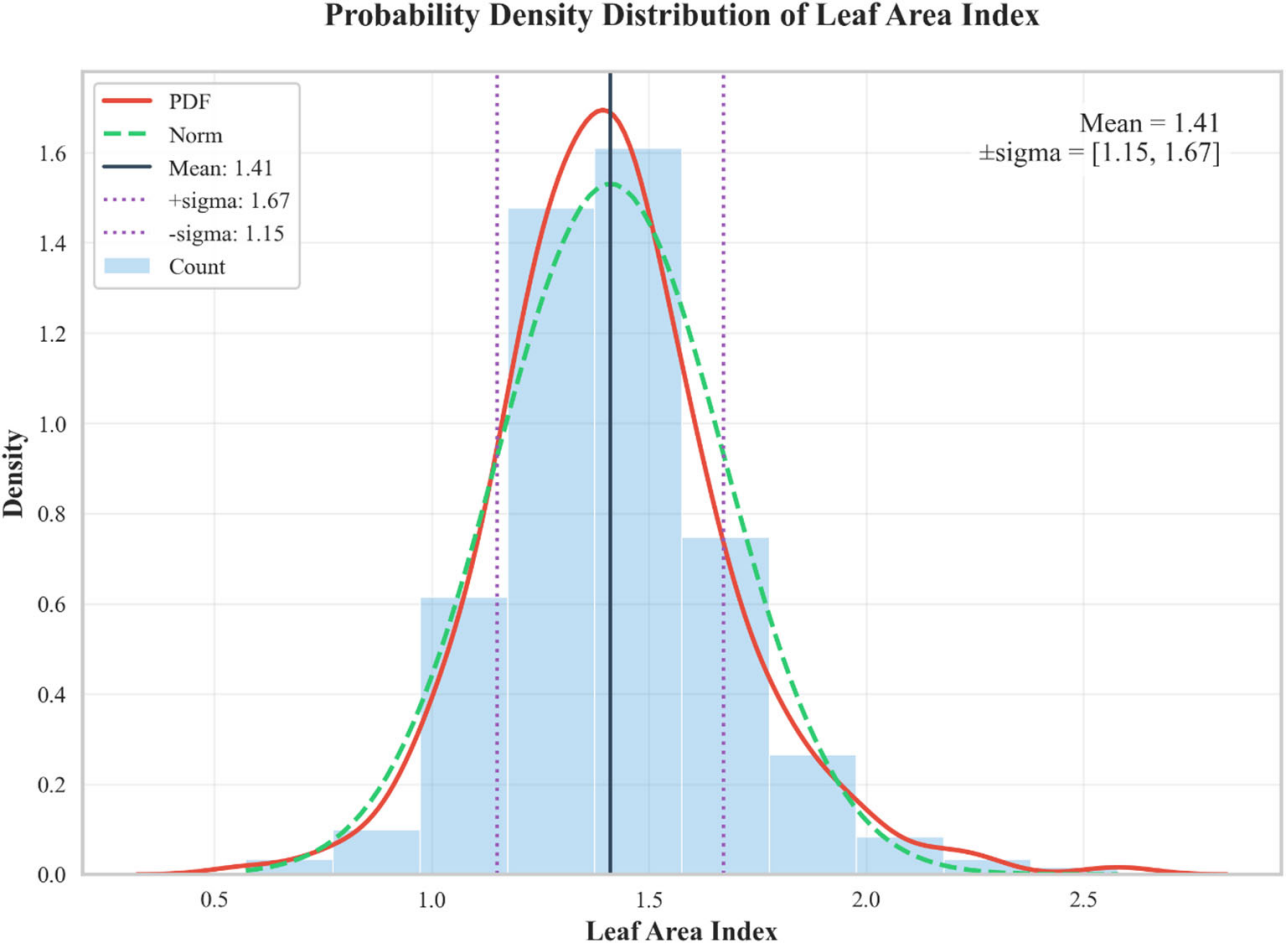
Histogram of LAI. The green dashed line represents a normal distribution fit, the red curve indicates the probability density function (PDF) fit, the purple dashed line shows data within one standard deviation, and the black solid line represents the mean value.

From August to September 2024, during three distinct growth stages—V6 (sixth trifoliate leaf), R1 (beginning bloom), and R3 (beginning pod)—the LAI of each soybean variety was measured using an AccuPAR LP-80 Plant Canopy Analyzer (Decagon Devices Inc., Pullman, WA, USA). Measurements were conducted between 10:00 a.m. and 1:00 p.m. local time on clear, calm days under stable sunlight conditions to ensure consistent illumination and measurement accuracy. For each of the 100 soybean varieties, the experimental plot was divided into four sampling zones, and LAI was measured once per zone to calculate the average LAI, resulting in 400 measurements per growth stage. Measurements were conducted at three different growth stages, yielding a total of 1,200 LAI measurements across all varieties, zones, and growth stages. **Figure 3** shows the distribution of LAI measurements across the three periods. These measurements were performed concurrently with the UAV flights.

### 2.4 UAV data collection

**Figure 4:**
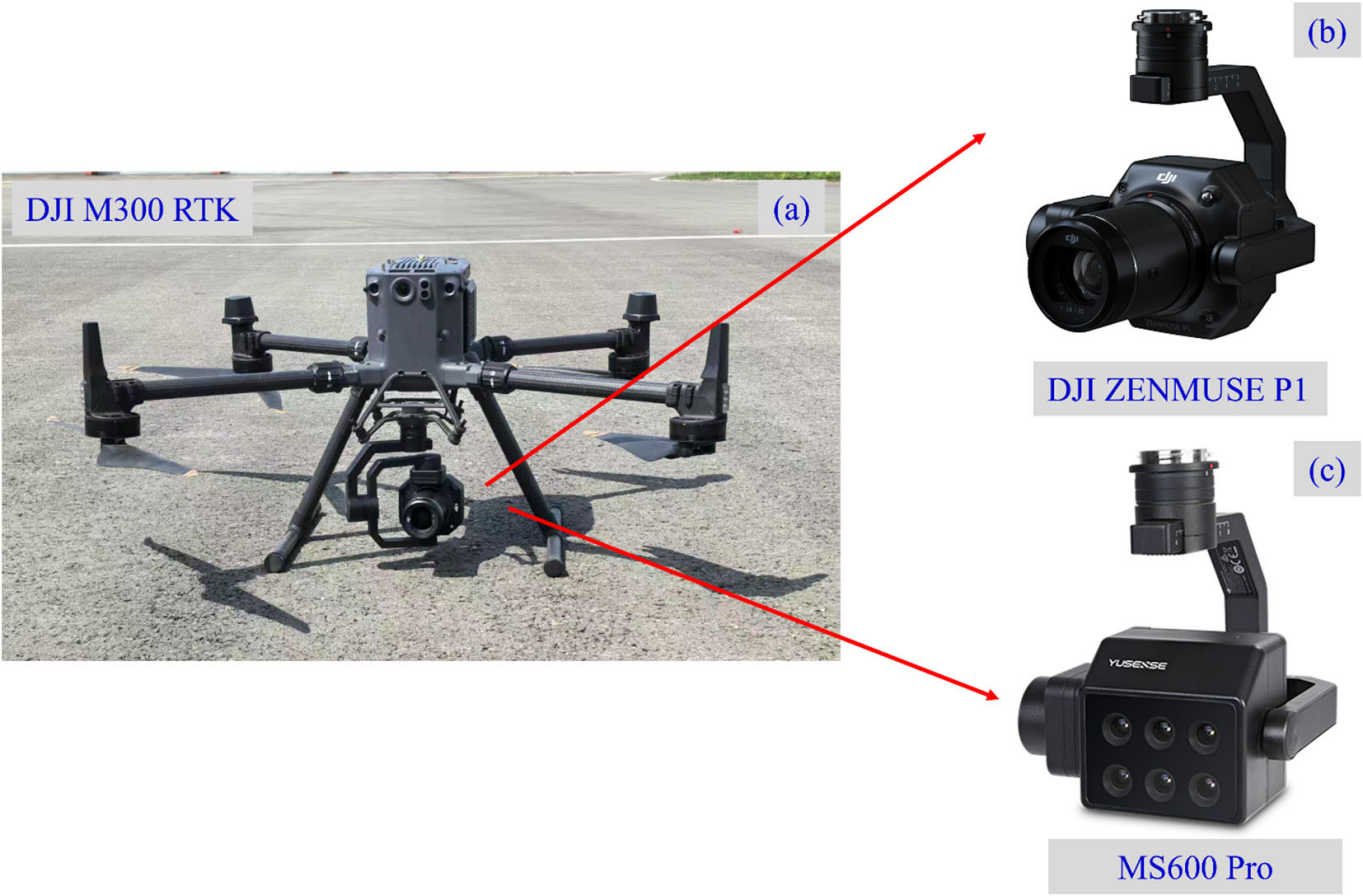
UAV-based crop phenotyping observation platform. **(a)** DJI M300 RTK UAV; **(b)** Zenmuse P1 RGB camera; **(c)** MS600 Pro MS camera.

In this research, UAV data were collected using a DJI M300 drone (Shenzhen, China) equipped with a DJI ZENMUSE P1 camera and an MS600 Pro MS camera (Qingdao UVS Technology Co., Ltd), as shown in **Figure 4**. The MS camera features six monochromatic channels: near-infrared (NIR, 840 ± 15 nm), red edge at 750 nm (RE750, 750 ± 5 nm), red edge at 720 nm (RE720, 720 ± 5 nm), red (R, 660 ± 11 nm), green (G, 550 ± 14 nm), and blue (B, 450 ± 15 nm). The DJI M300 includes an integrated Global Navigation Satellite System (GNSS) module for recording the geolocation data of the imagery. The main specifications of the multispectral camera, including each band and its central wavelength range, are summarized in **Table 1**, providing a clear reference for subsequent vegetation index calculations and image analysis. Data collection flights were conducted on August 7, August 23, and September 11, 2024, between 10:00 a.m. and 1:00 p.m. local time, under low wind and cloud-free conditions. The flight altitude was set at 15 m, 30 m, 45 m, and 60 m.

**Table 1:** Spectral bands and wavelength ranges of the MS600 Pro multispectral camera.

Band no.	Band name	Central wavelength (nm)	Bandwidth (nm)
1	Blue	475 ± 16	32
2	Green	560 ± 16	32
3	Red	668 ± 16	32
4	Red Edge	717 ± 10	40
5	NIR	842 ± 26	52
6	NIR2	900 ± 20	40

### 2.5 Image preprocessing

For MS images, standard reflectance calibration and radiometric correction were performed using Yusense Map software (Qingdao, China) to ensure comparability of images collected at different growth stages. The raw MS images were subsequently stitched together. To eliminate soil pixels, the Normalized Difference Vegetation Index (NDVI) for each pixel was calculated, and pixels with an NDVI value below 0.2 were identified as soil and excluded from the orthoimages.

Following the experimental layout’s dimensions, images were batched by varietal category to produce corresponding RGB and MS images. As previously described, images were categorized based on collection altitude and time, resulting in a total of 2400 images (1200 RGB images and 1200 MS images) for model construction.

### 2.6 Image SR and feature extraction

Building on the regionally segmented RGB images, this study explored four representative image SR techniques to enhance the resolution and quality of soybean leaf images: Super-Resolution Convolutional Neural Network (SRCNN), Enhanced Deep Super-Resolution Network (EDSR), Real-Enhanced Super-Resolution Generative Adversarial Network (Real-ESRGAN), and Swin Transformer for Image Restoration (SwinIR). These methods span from early con-volutional neural networks (CNNs) to recent generative adversarial networks (GANs) and Transformer architectures, providing a broad representation and complementarity suited to the diversity and complexity of soybean leaf images. SRCNN serves as a baseline model with a simple structure and high computational efficiency. EDSR is noted for its superior performance in various SR tasks, particularly excelling in the restoration of image details (Bashir et al., 2021). Real-ESRGAN effectively addresses real-world image degradation issues, enhancing texture details and improving visual quality. SwinIR integrates local and global attention mechanisms, effectively capturing both local and global features of images, especially those with complex textures (Ali et al., 2023). This study introduces these four distinct architectural SR algorithms to identify the most suitable SR methods for this research context.

These four SR methods were chosen over other alternatives because they collectively represent the main categories and technical milestones in SR development—namely, the foundational CNN-based models (SRCNN, EDSR), the GAN-based generative model (Real-ESRGAN), and the Transformer-based attention model (SwinIR). This selection ensures a comprehensive coverage of methodological diversity, allowing the study to evaluate performance differences across architectures with varying capacities for detail enhancement, texture restoration, and noise suppression. Such representativeness makes them particularly suitable for assessing SR performance under the complex texture and illumination conditions of soybean leaf imagery.

This study therefore employs these four distinct SR architectures to identify the most suitable approach for high-quality soybean leaf image reconstruction and analysis. To provide a clear overview, the main characteristics of these four SR methods are summarized in **Table 2**.

**Table 2:** Comparison of the main characteristics of the four SR methods used in this study.

Method	Model type	Core architecture	Key strength	Year
SRCNN	CNN-based	Shallow 3-layer convolutional network	Pioneer of deep learning SR, simple and effective	2014
EDSR	CNN-based (Residual)	Deep residual blocks without batch normalization	High reconstruction accuracy and robustness	2017
Real-ESRGAN	GAN-based	Enhanced ESRGAN with improved degradation modeling	Realistic detail and texture generation	2021
SwinIR	Transformer-based	Swin Transformer with local and global attention	Excellent texture recovery and feature preservation	2021

RGB images provide high-resolution spatial information, capturing the morphological features of soybean leaves such as edges, textures, and color variations. MS images, spanning multiple bands from visible light to near-infrared, offer richer spectral information. This is especially true in the near-infrared band, where higher vegetation reflectance can effectively indicate the physiological characteristics and health status of the leaves. Utilizing these sources, this study extracted 15 features from RGB image datasets and 15 features from MS images.

To accurately estimate the soybean LAI, we adopted a multi-source remote sensing data fusion method. This approach combines the morphological features from RGB images with the spectral features from MS images to create an integrated RGB+MS dataset. By leveraging the sensitivity of different bands to vegetation characteristics, this method enhances the accuracy and stability of LAI estimation. Consequently, the combined dataset features 15 morphological indicators from RGB and 15 spectral indicators from MS, totaling 30 indicators. **Table 3** summarizes the features used for modeling, which were extracted from the RGB, MS, and combined RGB+MS datasets. The full names and calculation formulas of these features are provided in **Appendix Table A1**.

**Table 3:** UAV image modeling features included in this study.

Sensors	Modeling features
RGB	R_Mean, G_Mean, B_Mean, Variance, GRVI, MGRVI, RGBVI, ExG, BGI, BRI, GRI,GLI,VARI,NGRDI,TGI
MS	MSAVI2, RVI, NDVI, EVI, RERVI, SAVI, DVI, OSAVI, GVI, TCARI, NDRE, ARI1, CRI2, GNDVI, RDVI
RGB+MS	R_Mean, G_Mean, B_Mean, Variance, GRVI, MGRVI, RGBVI, ExG, BGI, BRI, GRI, GLI, VARI, NGRDI,MSAVI2, RVI, NDVI, EVI, RERVI, SAVI, DVI, OSAVI, GVI, TCARI, NDRE, ARI1, CRI2, GNDVI, RDVI

### 2.7 LAI regression models

In this research, two machine learning approaches—Random Forest (RF) and Extreme Gradient Boosting (XGBoost)—were applied to construct models for soybean leaf area index estimation. RF (Natekin and Knoll, 2013), a well-known ensemble algorithm, enhances the predictive power of individual decision trees by aggregating them into a strong learner. Compared with traditional single-tree models, RF reduces the risk of overfitting and provides stable regression outcomes through the collective contribution of multiple weak learners.

XGBoost, on the other hand, has gained wide attention in recent years, particularly within agricultural studies. Unlike RF, it introduces a regularization term (Ω) to penalize model complexity, which effectively controls overfitting and improves robustness when handling high-dimensional datasets. Beyond accuracy, XGBoost is also capable of efficiently managing sparse data and supports distributed parallel training, making it highly suitable for large-scale regression problems (Karthikeyan and Mishra, 2021).

To maximize predictive performance, a systematic hyperparameter tuning procedure was adopted. Among the parameters, the learning rate was given particular importance since it determines the step size of error correction during training, directly influencing both convergence stability and final model quality. A two-stage grid search combined with 5-fold cross-validation was carried out: the initial stage identified suitable parameter ranges, followed by a refined search for optimal values. Model performance under each setting was assessed using the loss function, where lower values indicated improved prediction accuracy.

Given the stochastic nature of RF and XGBoost, the training and validation process was repeated 10 times for each hyperparameter configuration to account for variability due to random initialization and data sampling. The performance metrics, including loss and prediction accuracy, were averaged across these repetitions to provide robust estimates, and the standard deviations were calculated to quantify variability. This procedure ensures that the reported model performance is statistically reliable and not driven by random fluctuations in individual runs.

This rigorous optimization process notably enhanced both computational efficiency and generalization ability of the models. The joint use of grid search and cross-validation is a widely accepted practice for reducing overfitting and improving reliability in complex regression or classification tasks. For example, Adnan et al. (2022) reported significant improvements in model accuracy when employing this strategy in combination with adaptive boosting techniques.

### 2.8 Feature selection method

In machine learning, feature selection is the process of identifying a subset of the most valuable features from an original set for prediction tasks, aiming to eliminate redundant and irrelevant features, simplify the model structure, improve training efficiency, and enhance generalizability. Redundant features are highly correlated with other features, providing information that may already be contained elsewhere, while unrelated features have little or no significant impact on the target variable. Retaining such features not only increases model complexity but can also introduce noise, leading to overfitting and reduced performance on new data.

In this study, a model-based feature selection method was adopted to improve the predictive performance of the model and reduce computational burden. Specifically, the SelectFromModel(SFM) class provided by the scikit-learn library in Python version 3.6.13 (https://scikit-learn.org) was utilized for feature selection.

This method trained a basic estimator (such as a linear model or tree model), which evaluated the importance of each feature using its provided feature importance index (Raschka, 2018).Subsequently, based on the set threshold, features with importance higher than the threshold were selected to form a new feature subset. This approach integrates the feature selection process into model training, avoiding the need to train multiple models repeatedly, thus enhancing processing efficiency.

In this study, ‘mean’ was selected as the threshold, which means retaining features with importance scores higher than the average importance of all features. This strategy can be beneficial in removing redundant or irrelevant features. Moreover, the strategy can also reduce the risk of overfitting and improve the model’s generalizability.

By applying the SelectFromModel method, the most contributing features to the inversion of soybean LAI were selected from the original feature set. These features can offer a more streamlined and efficient data foundation for subsequent modeling and analysis.

### 2.9 Evaluation index

For the verification of the LAI model’s estimation accuracy, 70% of the samples and observed leaf age were randomly selected for training the model. While the remaining 30% of the samples were used to validate the performance and accuracy of the estimation model. Coefficient of determination R^2^ (Equation 1), root mean square error (Equation 2), relative RMSE, and mean absolute error were employed to assess the stability of the model in various aspects. The calculation formula is formulated as follows:

(1) $$R^{2}=\frac{{\sum_{i=1}^{n}\left({\hat{y}}_{i}-\bar{y}\right)}^{2}}{{\sum_{i=1}^{n}\left(y_{i}-\bar{y}\right)}^{2}}$$

(2) $$\text{RMSE}=\sqrt{\frac{1}{n}{\sum_{i=1}^{n}\left(y_{i}-{\hat{y}}_{i}\right)}^{2}}$$

(3) $$\text{rRMSE}=\frac{\sqrt{\frac{1}{n}{\sum_{i=1}^{n}\left(y_{i}-{\hat{y}}_{i}\right)}^{2}}}{\frac{1}{n}{\sum_{i=1}^{n}y}_{i}}$$

(4) $$\text{MAE}=\frac{1}{n}\sum_{i=1}^{n}|y_{i}-{\hat{y}}_{i}|$$

Among them, *y_i_* denotes the total number of samples. *y_i_* and ${\hat{y}}_{i}$ represent the LAI of the measured and estimated values of the samples, respectively. $\bar{y}$ is the average value of measured LAI. *R*^2^indicates the correlation between measured results and estimated results. RMSE and rRMSE (Equation 3) represent the loss and loss rate between measurement results and estimated results. MAE (Equation 4) represents the absolute value between measured result and estimated result. The lower the RMSE or rRMSE, the better the model estimation accuracy.

To comprehensively assess the quality of image SR reconstruction, two widely used objective evaluation metrics were adopted: Peak Signal to Noise Ratio (PSNR, Equation 5) and Structural Similarity Index (SSIM, Equation 6).

(5) $$\text{PSNR}=10\cdot \log_{10}(\frac{{\text{MAX}}_{I}^{2}}{\text{MSE}})$$

(6) $$\text{SSIM}(x,y)=\frac{\left(2\mu_{x}\mu_{y}+C_{1})(2\sigma_{xy}+C_{2}\right)}{\left(\mu_{x}^{2}+\mu_{y}^{2}+C_{1})(\sigma_{x}^{2}+\sigma_{y}^{2}+C_{2}\right)}$$

PSNR is a commonly utilized metric for assessing the quality of image reconstruction, primarily used to evaluate the differences between reconstructed images and original images, based on mean squared error (MSE). Here, MAX*_I_* represents the potential maximum pixel value in an image, which for an 8-bit image is MAX*_I_* = 255. PSNR is measured in decibels (dB), with higher values indicating closer proximity of the reconstructed image to the original, denoting better quality. SSIM is an image quality metric more aligned with human visual perception, assessing similarity between two images across three dimensions: luminance, contrast, and structure. Here, *μ_x_* and *μ_y_* are the mean luminance values of images *x* and *y*, respectively, $\sigma_{x}^{2}$ and $\sigma_{y}^{2}$ are the variances, *σ_xy_* is the covariance, and *C*_1_ and *C*_2_ are small constants used to stabilize the denominator. The SSIM value ranges from [–1, 1], with values closer to 1 indicating greater similarity between the two images.

## 3 Results

### 3.1 Super resolution results

**Figure 5:**
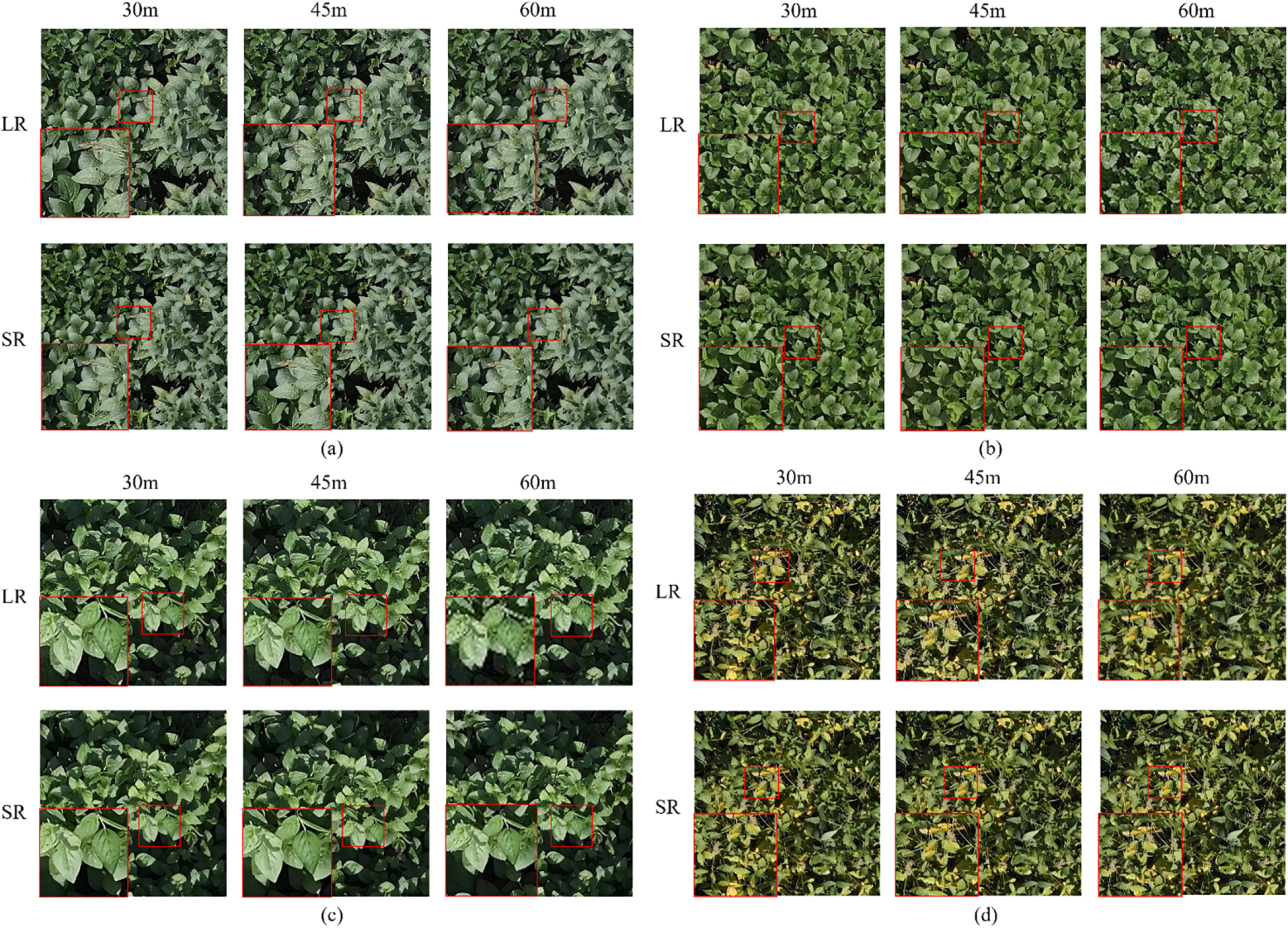
Results of resolution enhancement using SRCNN **(a)**, EDSR **(b)**, Real-ESRGAN **(c)**,and SwinIR **(d)**. Each panel presents the reconstructed soybean canopy images after applying the corresponding model. SRCNN **(a)** and EDSR **(b)** improve overall clarity and detail definition compared to the original low-resolution input. Real-ESRGAN **(c)**, based on a generative adversarial network (GAN), effectively enhances texture realism and reduces artifacts. SwinIR **(d)**, employing a Transformer architecture, produces visually sharp and natural results with well-preserved canopy structures. Overall, both the GAN- and SwinIR-based methods exhibit superior enhancement quality compared with the other approaches.

The outcomes of applying four SR techniques to enhance the resolution of RGB images are depicted in **Figure 5**, whereas **Table 4** presents a comparative analysis of the metrics before and after the application of these SR techniques. The data clearly indicates that the best SR effects were achieved at a 30-meter altitude, with an average PSNR of 28.09, surpassing the results at 45-meter (26.76 PSNR) and 60-meter (26.12 PSNR) altitudes. This suggests that the effectiveness of SR techniques diminishes with increasing flight altitude, with minimal enhancement in image quality observed at a higher altitude of 60 m. Among the four SR methods evaluated, SwinIR demonstrated superior performance, achieving enhanced results in both PSNR and SSIM compared to the other three methods. This underscores the advantages of SwinIR’s hierarchical window-based attention mechanisms in processing and restoring the edges of soybean leaves.

**Table 4:** Image metrics obtained from four SR methods.

Altitude	Evaluation index	SRCNN	EDSR	Real-ESRGAN	SwinIR
30 m	PSNR	27.95	28.01	28.10	28.33
	SSIM	0.9295	0.9327	0.9363	0.9460
45 m	PSNR	26.28	26.52	26.97	27.25
	SSIM	0.9407	0.9434	0.9498	0.9579
60 m	PSNR	25.77	25.94	26.25	26.50
	SSIM	0.9427	0.9483	0.9565	0.9624

In general, a higher PSNR value (typically above 30 dB for high-quality natural images) indicates better reconstruction fidelity, whereas an SSIM value closer to 1.0 reflects greater structural similarity between the reconstructed and reference images. Therefore, the observed PSNR and SSIM values in this study suggest that SwinIR achieved relatively higher image fidelity and structural consistency compared with the other SR methods.

### 3.2 Feature selection results

A feature dataset comprising all input features was constructed based on each cropped plot image. To mitigate the impact of inter-feature correlation, feature selection was performed using the SelectFromModel (SFM) method described in Section 2.8.In this study, two machine learning methods were utilized; therefore, RF and XGBoost models were employed for feature selection. Table displays the subsets of features selected by the SFM method from three types of input datasets, all chosen from images at a 15-meter altitude without SR.

**Figure 6:**
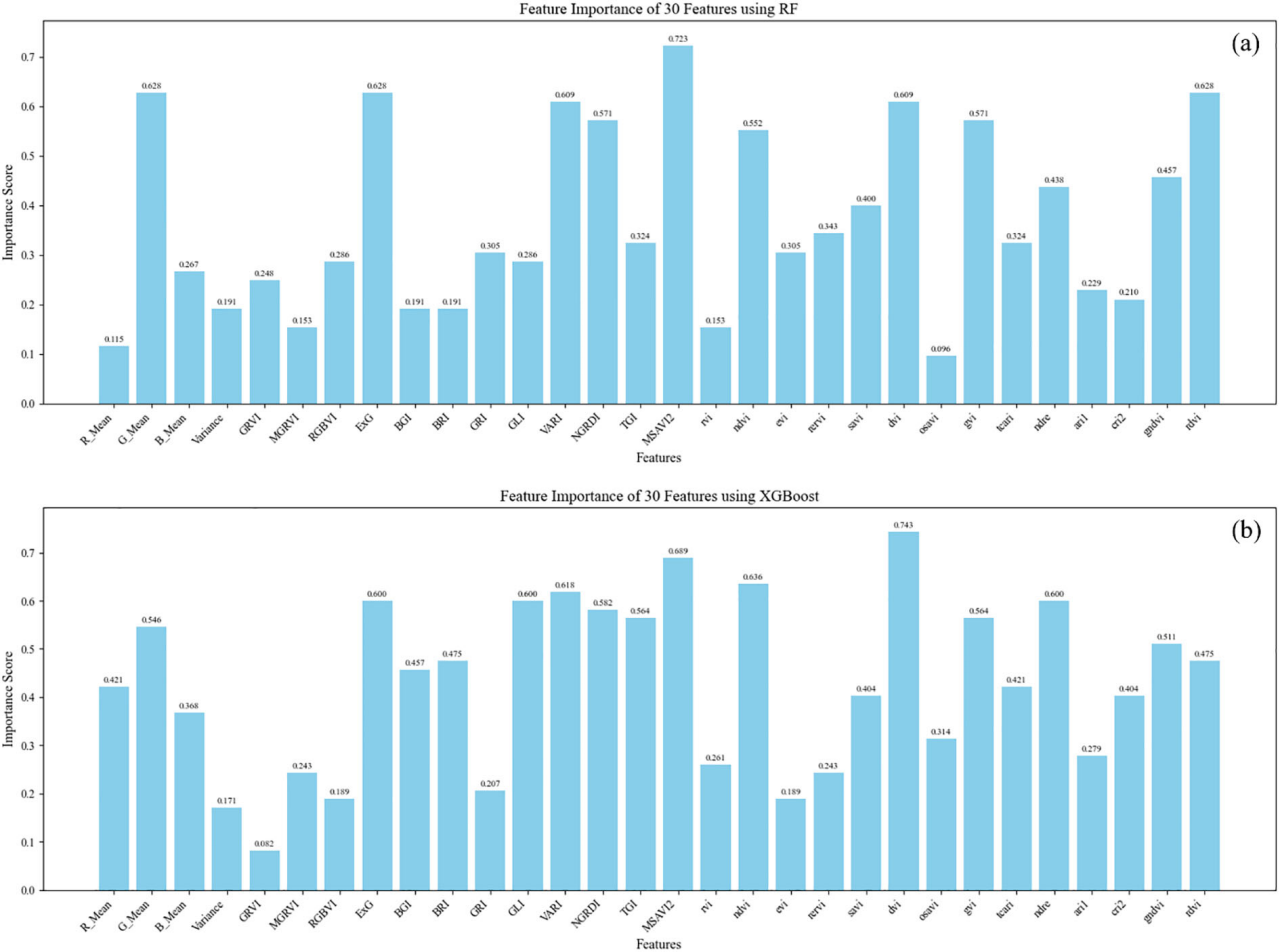
Feature importance of 30 selected features evaluated using two tree-based ensemble methods: **(a)** Random Forest (RF) and **(b)** XGBoost. The importance scores shown reflect the relative contribution of each feature to the model’s predictive performance. Differences between RF and XGBoost reflect variations in how each method assesses feature relevance.

The data in **Table 5** reveals that among the two SFM methods, several features such as ExG, Variance, VARI, NIR, and G_Mean were consistently selected across the three input datasets, indicating a strong correlation with the LAI. Additionally, indices such as MSAVI2 and NDVI were prioritized in the modeling workflow due to their established sensitivity to canopy structure and leaf area, which makes them particularly informative for estimating LAI. The relative contribution of each feature to the predictive performance of the RF and XGBoost models is illustrated in **Figure 6**, where the importance scores reflect the influence of each feature in the modeling process. Overall, it is evident that both the textural and spectral characteristics of soybean leaves are pertinent to the construction of the model.

**Table 5:** Feature combinations selected from the complete set of input features from three sensors, using different feature selection methods.

Feature selection sensors	SFM_RF	SFM_XGBoost
RGB	ExG, VARI, GRVI, Variance,G_Mean, TGI, RGBVI	ExG, VARI, GRVI, Variance,G_Mean, TGI
MS	NDVI, NDRE, EVI, SAVI, NIR, DVI, MSAVI2	NDVI, NDRE, EVI, SAVI, NIR, RVI, MSAVI2
RGB+MS	NDVI, NDRE, EVI, ExG, VARI, SAVI, MSAVI2,TGI, Variance,G_Mean	NDVI, NDRE, EVI, ExG, VARI, SAVI, MSAVI2,GRVI, Variance,G_Mean

### 3.3 Modeling and validation of LAI estimation

#### 3.3.1 Evaluation of different sensors and regression models

**Table 6** presents the validation statistics for various input combinations, specifically the coefficients of determination (R^2^), RMSE, and relative RMSE. All data within the table were derived from images taken at an altitude of 15 m. It is evident that among the three feature input combinations, the XGBoost model exhibited the most proficient performance in regressions related to leaf area, achieving the highest overall precision. When considering the source of input variables, the combination of RGB and MS sensors provided the most favorable outcomes. For the XGBoost model, the R² varied between 0.8703 and 0.9173, while rRMSE ranged from 4.26% to 6.17%. These metrics confirm the robustness of the models developed in this study. Concurrently, the use of XGBoost for feature selection and model construction, coupled with the fusion of multiple data sources (RGB+MS), emerged as the most precise approach.

**Table 6:** Validation statistics for different soybean LAI estimation models.

Feature selection and modeling approach	Metrics	RGB	MS	RGB+MS
All input features_RF	R^2^	0.8553	0.8401	0.8635
	RMSE	0.0926	0.0952	0.1074
	rRMSE	7.87%	10.21%	5.86%
All input features_XGBoost	R^2^	0.8592	0.8417	0.87014
	RMSE	0.0845	0.0917	0.0986
	rRMSE	7.24%	9.86%	5.28%
SFM_RF	R^2^	0.8643	0.8446	0.8824
	RMSE	0.0705	0.0725	0.0685
	rRMSE	6.74%	11.02%	6.19%
SFM_XGBoost	R^2^	0.8703	0.8558	0.9173
	RMSE	0.0732	0.0903	0.0478
	rRMSE	5.25%	9.17%	4.26%

Highlighted statistics represent the most accurate estimations of LAI by each sensor data source.

#### 3.3.2 Performance of different LAIs on model accuracy

**Figure 7:**
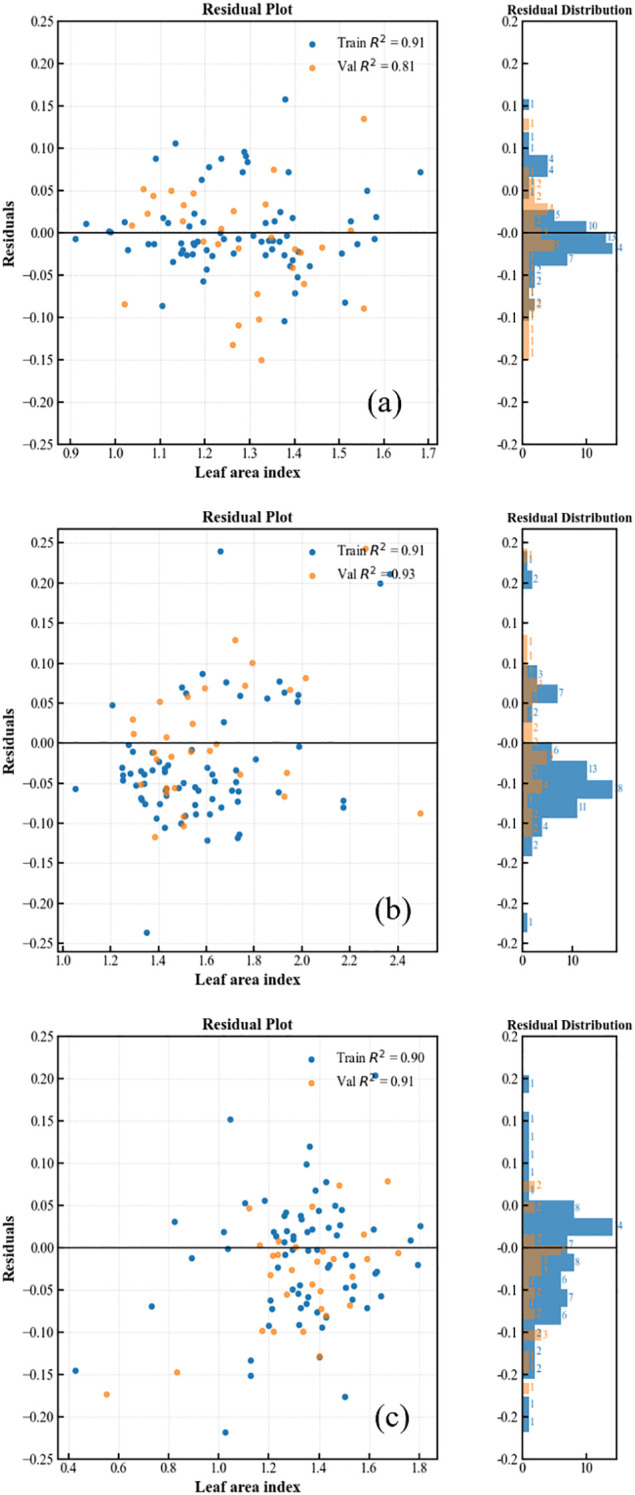
Residual distribution of the XGBoost model for RGB sensor **(a)**, MS sensor **(b)**, and RGB+MS sensor **(c)**. Train: Training dataset. Val: Validation dataset.

In this section, the performance of the XGBoost model combined with multisource information fusion (RGB+MS) was evaluated across different periods for leaf area estimation. **Figure 7** displays the final evaluation results. In each panel, the scatter plot on the left plots the estimated leaf area (x-axis) against the residuals, which are the differences between the estimated and true leaf areas (y-axis). On the right, a histogram illustrates the distribution of these residuals. The analysis indicates that the model adapts well to leaf area estimation, with absolute residuals less than 0.25, and the residuals across three dates approximating a normal distribution.

#### 3.3.3 Evaluation of various SR techniques

**Figure 8:**
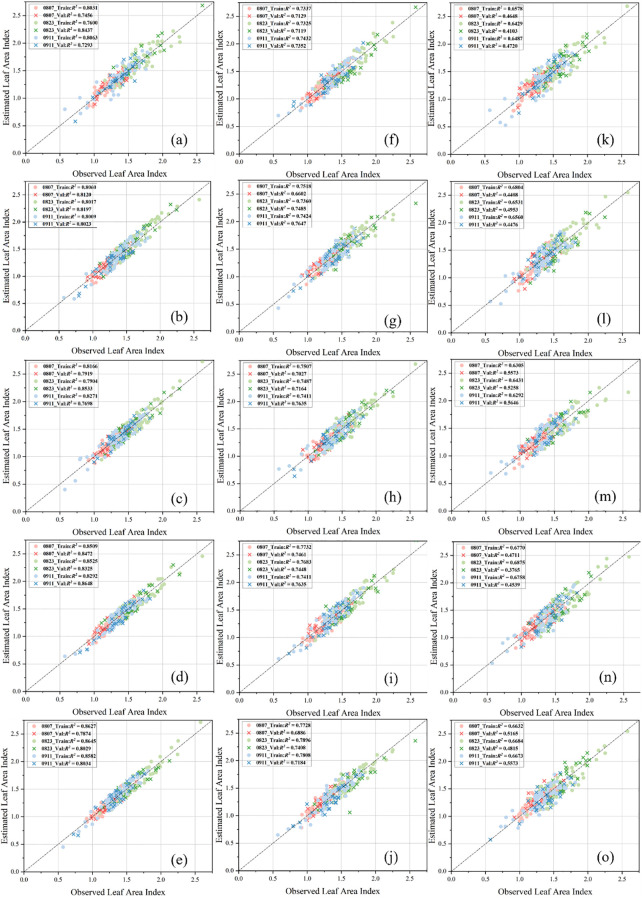
Relationship between the estimated and measured LAI across three different altitudes and four SR methods within both training and validation datasets. At 30-meter altitude: **(a)** without SR, **(b)** using SRCNN, **(c)** using EDSR, **(d)** using Real-ESRGAN, **(e)** using SwinIR. At 45-meter altitude: **(f)** without SR, **(g)** using SRCNN, **(h)** using EDSR, **(i)** using Real-ESRGAN, **(j)** using SwinIR. At 60-meter altitude: **(k)** without SR, **(l)** using SRCNN, **(m)** using EDSR, **(n)** using Real-ESRGAN, **(o)** using SwinIR. The color coding indicates the dates of LAI estimation: red for August 7, green for August 23, and blue for September 11. The data points in the figures are differentiated into training and validation datasets, as indicated by distinct shapes.

This study employed four distinct SR methods—SRCNN, EDSR, Real-ESRGAN, and SwinIR—to assess their efficacy in estimating the LAI within a regression model. The original altitudes evaluated were 30 m, 45 m, and 60 m. For each altitude, the LAI was enhanced using the aforementioned SR techniques, maintaining identical input features as those used at a baseline altitude of 15 m. **Figure 8** illustrates the relationship between the measured LAI and the estimated LAI at an altitude of 30 m, following optimization with the four algorithms. It was observed that the introduction of SR techniques significantly enhanced the overall precision of the model. Specifically, the SRCNN and EDSR methods showed improvements in their R^2^ values compared to the original 30-meter images, although the increases were not substantial. Conversely, the Real-ESRGAN and SwinIR methods demonstrated a noticeable rise from an R^2^ of approximately 0.80 to around 0.86. **Figure 8** also depicts the relationships between the LAI values at an altitude of 45 m after optimization with the four algorithms. At this altitude, the application of SR methods continued to positively influence the model’s precision. The performance enhancements with SRCNN and EDSR were similar to those at 30 m, whereas the Real-ESRGAN and SwinIR methods did not show as significant an increase as seen in the 30-meter scenario, indicating a diminishing return in model precision enhancement. Furthermore, **Figure 8** displays the relationship between the LAI values at 60 m following the application of the four algorithms. At this altitude, none of the SR methods demonstrated a noticeable change in model precision compared to the unprocessed data, suggesting that SR techniques did not contribute to precision enhancement under these conditions.

## 4 Discussion

### 4.1 Evaluation metrics for SR algorithms

This study employs the PSNR and SSIM indices to evaluate the SR effect. Despite their widespread use in image quality assessment, PSNR and SSIM exhibit numerous limitations in the agricultural sector. They struggle to reflect the semantic fidelity of key agricultural features within images, respond sensitively to changes in critical areas, and are highly sensitive to environmental variations (Hore and Ziou, 2010; Nigam and Jain, 2020). Therefore, in agricultural image processing, a comprehensive evaluation should incorporate perceptual indices or task-related metrics. This study evaluates the effect of SR based on the accuracy of the final LAI evaluation.

### 4.2 Selection of data features for LAI evaluation

In **Table 6**, the model performance was evaluated using the coefficient of determination (R²), RMSE, and relative RMSE (rRMSE). These three indicators were selected because they collectively assess both the accuracy and relative deviation of model predictions, which are essential for comparing different feature combinations and model configurations. In contrast, the mean absolute error (MAE) was reserved for analyzing LAI estimation performance across different growth stages (Section 4.4), where absolute differences provide more intuitive insights into temporal variations. MAE is often preferred in such analyses because it expresses the average absolute error in the same units as the original data, allowing more straightforward interpretation of deviations over time (Chai and Draxler, 2014).

These results underscore the importance of high spatial resolution in revealing more details and enhancing the accuracy of feature data, thereby confirming the significant role of SR techniques in this study. Previous studies have shown that the fusion of information from different sensors can significantly enhance crop growth monitoring and yield prediction. For example, incorporating both RGB and multispectral (MS) features leads to more accurate estimation of rice LAI (Yang et al., 2021).

Although **Table 6** indicates that the results using RGB data alone are better than those using MS data alone, MS data remains of great importance. The introduction of MS provides an additional dimension of information for estimating leaf age from a spectroscopic perspective. MS imagery can detect fine spectral variations related to distinct physiological components of leaves more effectively than RGB data, thanks to its narrowband sensitivity (Reddy and Pawar, 2020). MS information offers critical insights for tracking physiological, biochemical, and structural dynamics across crop growth stages (Zhao et al., 2023). Leaf spectral reflectance is influenced by factors such as chlorophyll concentration, equivalent water thickness, and leaf inclination angle, which vary notably over time (Zeng et al., 2022). As leaves mature, significant changes also occur in water content, pigment levels, and other chemical constituents (Sims and Gamon, 2002). These changes are effectively captured through spectral imaging and feature extraction, reflecting trends over time.

In addition, leaf spectral characteristics play a crucial role in estimating LAI, as they capture both biochemical and structural variations of the canopy. Spectral reflectance in the visible region, particularly in the red and green bands, is primarily influenced by pigment content. In contrast, reflectance in the near-infrared (NIR) region is affected by leaf internal structure and canopy density (Haboudane et al., 2004). A higher LAI typically corresponds to stronger absorption in the visible range and higher reflectance in the NIR range, reflecting the integrated effects of canopy structure and photosynthetic activity.

Therefore, incorporating spectral information from multiple sensors provides a more comprehensive representation of canopy characteristics. In this study, the multi-source data fusion approach (RGB + MS) enabled the joint use of spectral and structural features, effectively improving LAI estimation accuracy. This emphasizes the importance of combining spectral indices with textural and canopy-structural features in the inversion process, demonstrating the advantage of multi-source fusion for capturing LAI-related variability (Delegido et al., 2011).

**Figure 9:**
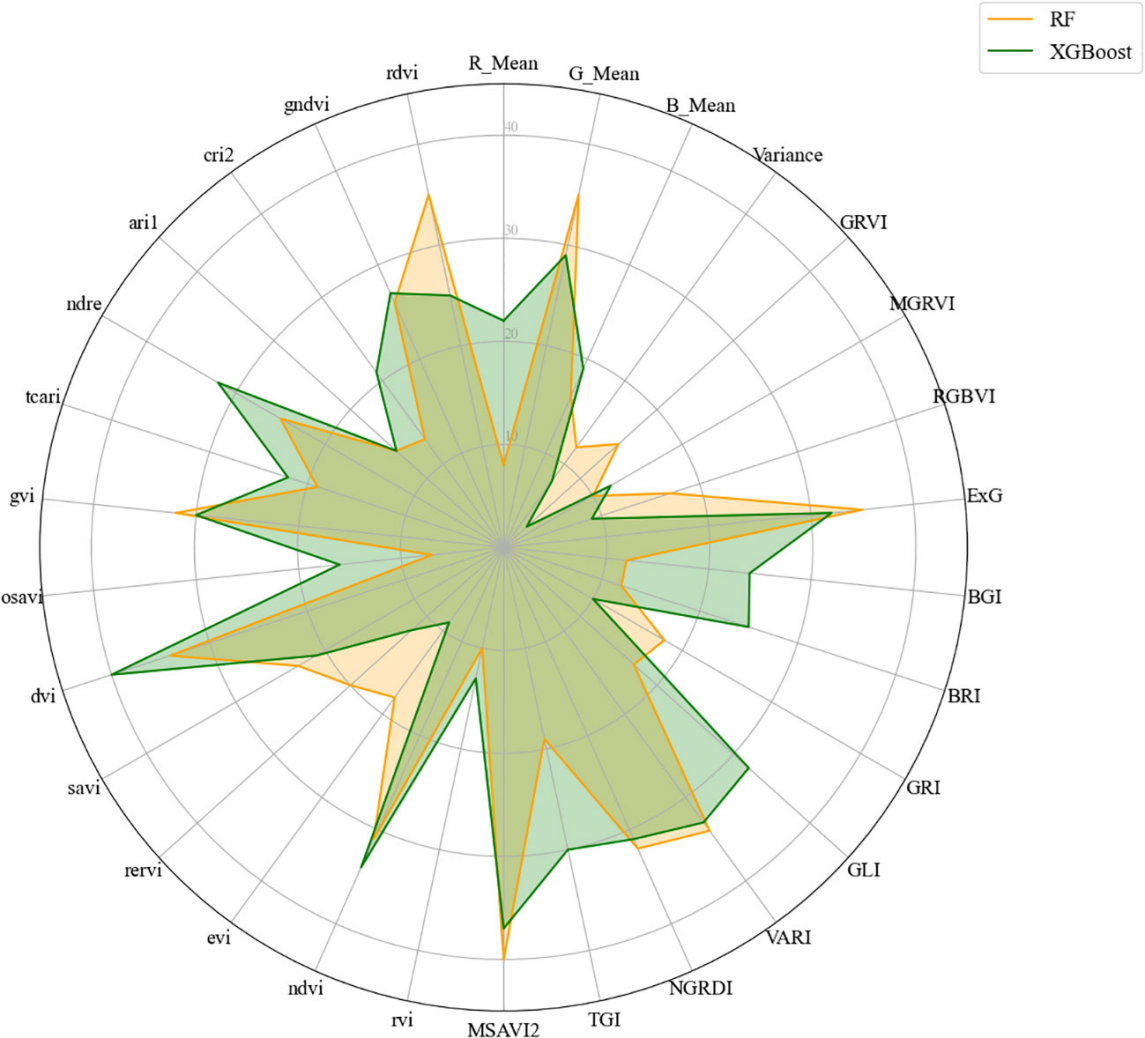
Image feature importance based on stepwise regression analysis.

The study investigated the impact of input image features on model performance by incrementally adding modeling features. As shown in **Figure 9**, in both the RF and XGBoost models, MSAVI2 and NDVI are the most influential features, underscoring their pivotal roles in predicting target variables. These vegetation indices collectively highlight the importance of spectral features in estimating LAI.

### 4.3 Analysis of SR algorithms

Previous research, as summarized in **Table 6**, indicates that the combination of the XGBoost model with multisource information fusion (RGB + MS) achieves the highest precision. Therefore, the subsequent analyses in this study are based on this combination. Following this determination, SR techniques were introduced during the data preprocessing phase. To investigate the enhancement effect of SR methods, flights were conducted at four different altitudes: 15 m, 30 m, 45 m, and 60 m, corresponding to spatial resolutions of 0.1875 cm, 0.375 cm, 0.5625 cm, and 0.75 cm, respectively. The results show that SR methods positively impact model precision. Among all four methods, Real-ESRGAN and SwinIR performed better than SRCNN and EDSR. This superiority is attributed to the intrinsic characteristics of agricultural images, such as fine textures (e.g., leaf details and crop textures) and repetitive leaf structures, which are prevalent in the RGB images used in this study.

The application of CNNs in image SR tasks was initiated by Dong et al. with the introduction of SRCNN, a three-layer, end-to-end convolutional network (Dong et al., 2015). This architecture demonstrated superior performance compared to traditional methods based on interpolation or sparse representation. With the rapid evolution of deep convolutional networks in the field of SR, EDSR, proposed in 2017, incorporated residual modules and deepened the network architecture to further improve SR performance (Lim et al., 2017). Within a CNN, a single convolutional kernel observes only a small local portion of the input image. Despite stacking multiple convolutional layers, the receptive field expands slowly. If a pixel is to perceive information from a distant location, it must traverse numerous layers, which can lead to attenuation or distortion of information during transmission. Furthermore, downsampling operations such as pooling, while capable of enlarging the receptive field, often result in loss of detail and texture degradation, making them unsuitable for restoring delicate structures in soybean RGB images. Consequently, employing CNNs to enhance the SR of soybean leaf images is not considered an appropriate approach.

**Figure 10:**
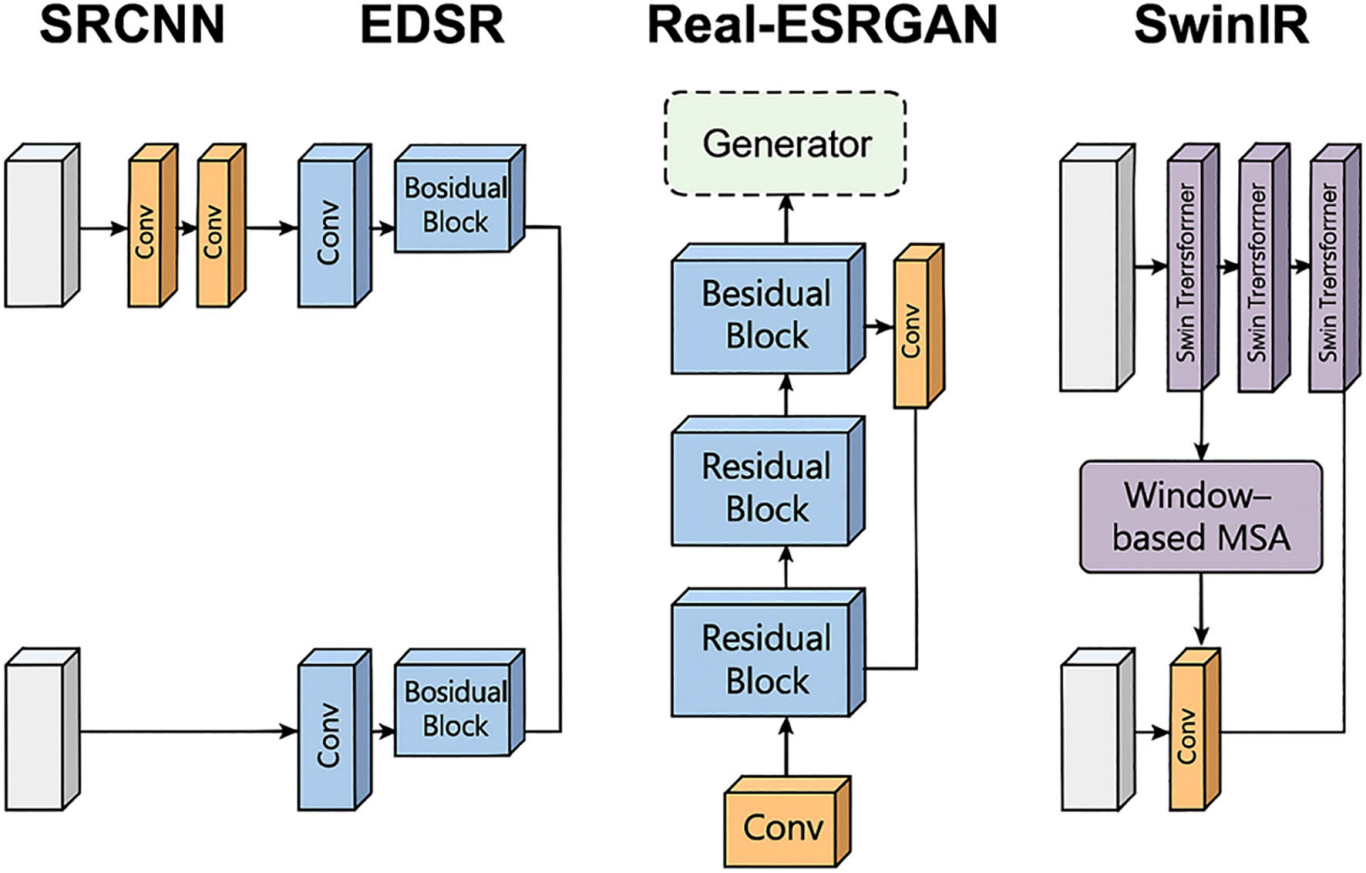
Comparison of network architectures for four SR methods.

Real-ESRGAN, introduced in 2021, builds upon EDSR by incorporating a more complex GAN structure and enhancing the training data degradation model to support more realistic low-quality inputs (Wang et al., 2021). SwinIR, also from 2021, is based on the Swin Transformer, a sliding window transformer that naturally excels at modeling long-distance dependencies and understanding distant texture correlations in images, which is particularly crucial in crop imaging (e.g., the directionality of leaf veins and textures) (Liang et al., 2021). **Figure 10** illustrates the structural differences among the four network architectures. SRCNN and EDSR rely solely on local convolution to interpret images, whereas Real-ESRGAN introduces a GAN structure that establishes an adversarial process between the generator and the discriminator. This process not only seeks numerical closeness but also aims to visually deceive the discriminator, thereby generating more natural images with high-frequency details (Zhang et al., 2023). SwinIR employs localized window attention and a sliding window mechanism, allowing for regional modeling while also capturing information from distant areas. Overall, Real-ESRGAN exhibits significant advantages in realistic degradation modeling and detail enhancement, while SwinIR achieves breakthroughs in feature modeling capability and distant texture reconstruction. Both methods surpass traditional CNN approaches in terms of visual perception quality and practical application results. These findings are consistent with the conclusions of this study, which indicate that models optimized with Real-ESRGAN and SwinIR exhibit higher precision than those using traditional CNN techniques. The differences in the network architectures of the four methods are shown in **Figure 10**.

### 4.4 Comparative analysis of soybean LAI across different time periods

**Figure 11:**
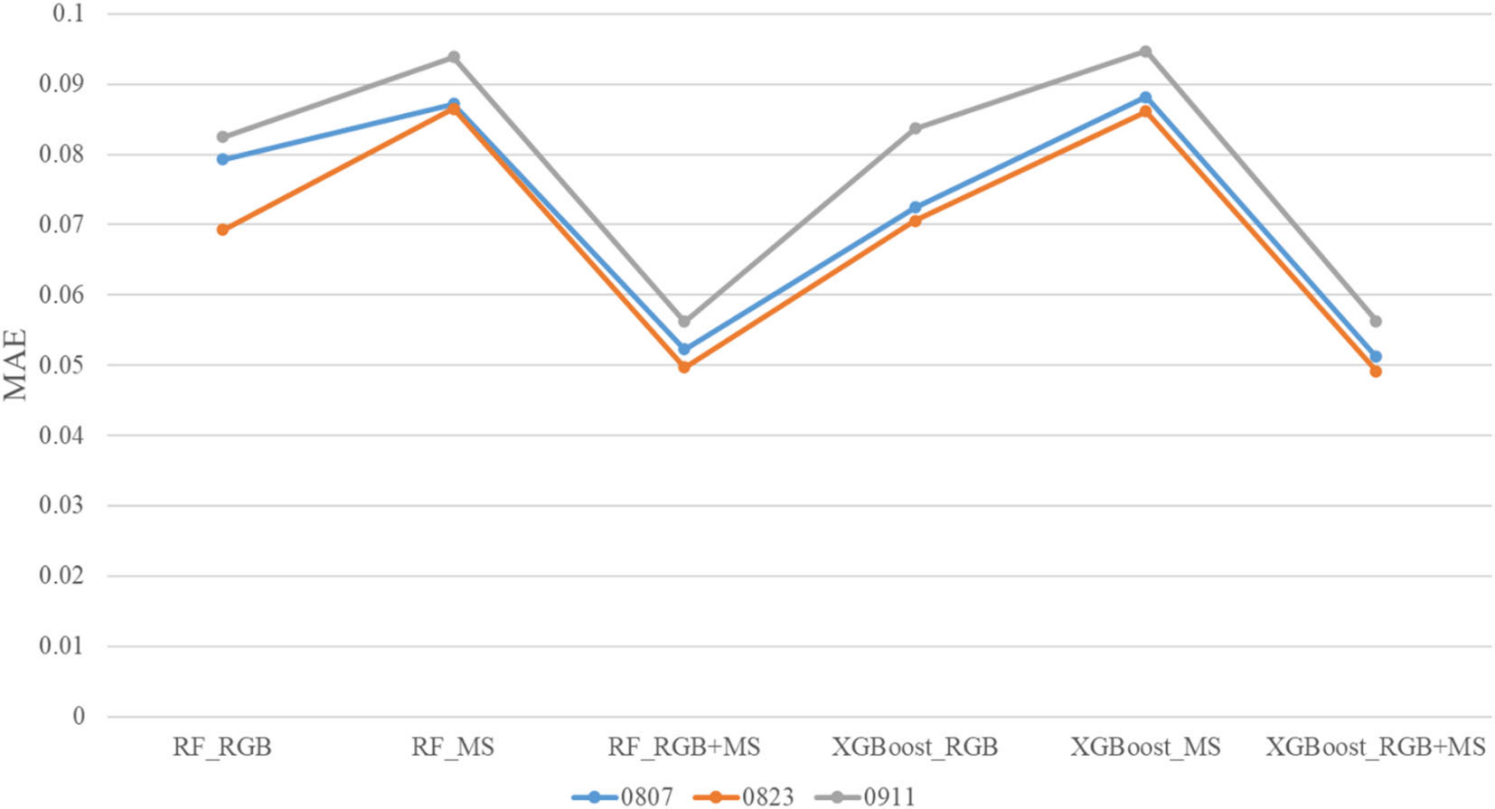
MAE of LAI estimates over three time periods.

To examine the influence of soybean LAI on the accuracy of estimation models, **Figure 11** illustrates the distribution of MAE for LAI estimates over three distinct time periods. Statistical analysis of the results revealed that the estimation model more accurately reflected LAI during August. However, estimation precision noticeably decreased during the late growth stage in September. One plausible explanation for this decline is the reduced sensitivity of vegetation indices, such as NDVI and EVI, to LAI. As the growth season progresses, leaf senescence and thinning occur, leading to a situation in which, despite a reduction in LAI, changes in NDVI and EVI become minimal, resulting in inaccurate model estimations. Leaf discoloration and the concomitant decrease in chlorophyll content cause vegetation indices to reflect not only leaf area but also information indicative of physiological aging. With reduced vegetation cover, more of the ground surface—including bare soil and dry grass—is exposed, mixing soil signals with those received by the sensors. Consequently, while vegetation indices may show a decline, substantial leaf area may still remain, causing the model to erroneously interpret a greater decrease in LAI. Moreover, spectral information primarily originating from leaves reduces the MAE of MS data used in LAI estimation.

To mitigate this limitation, future studies could incorporate vegetation indices that are more sensitive to senescence and pigment variation, such as the Red Edge Chlorophyll Index (CIred-edge) or the Normalized Difference Senescence Index (NDSI), which may enhance model robustness during late growth stages. In addition, although this study involved multiple soybean varieties, the current model’s generalizability across different genotypes, growth environments, and phenological stages remains to be further validated. Future research should therefore consider multi-environment and multi-temporal datasets to improve the universality and adaptability of the proposed model.

### 4.5 Limitations and future prospects

This study employed multi-source remote sensing imagery to estimate the LAI of soybean crops. The integration of SR techniques during the data preprocessing phase yielded favorable outcomes. However, the models’ ability to account for morphological and phenological variations among soybean crops at different growth stages and across varieties was not fully validated, which may limit the generalizability of the estimation models. Future research should incorporate multi-temporal and multi-varietal datasets to better capture these variations, thereby enhancing the model’s robustness and transferability under diverse field conditions. Compared to traditional destructive methods for collecting soybean LAI data, UAVs equipped with multiple sensors can rapidly and efficiently gather extensive crop information. This approach not only saves considerable labor and costs but also achieves higher data accuracy. Although the study covered several soybean varieties with identical sowing times, noticeable differences in growth conditions were observed. The methods developed here demonstrated strong adaptability in estimating LAI across different soybean varieties, indicating significant practical value and providing robust support for researchers.

The SR technique introduced at the data preprocessing stage was designed to maintain data accuracy despite higher UAV flying altitudes. It was demonstrated that within a specific altitude range, the SR method significantly enhanced model precision. The improvement in the resolution of RGB images at an altitude of 30 m was found to be comparable to that at the original flying altitude of 15 m. This highlights the considerable potential of deep learning models in agricultural surveillance. In future studies, additional modules could be integrated to further optimize the SR model, improving its ability to address texture and leaf-repetition issues in soybean imagery. Such advancements could inspire new approaches to enhance UAV data collection efficiency and model generalization across phenological stages and varieties.

## 5 Conclusion

This study aimed to estimate the LAI of soybeans using UAVs equipped with high spatial resolution RGB and MS imagery. We employed appropriate feature selection methods to enhance the efficiency of the modeling process. The study identified the most precise combinations of modeling techniques and datasets and subsequently developed a high-precision model for estimating the LAI.

Additionally, SR techniques were introduced during the data preprocessing stage to enhance the RGB image data before its incorporation into the model, resulting in higher precision outcomes. The key findings of this research are as follows:

1. The combination of features from both RGB and MS imagery was superior to using either type of data alone. Features derived from the MS data significantly enhanced model precision.
2. The use of feature selection methods improved the operational efficiency of the model. Among the models constructed in this study, namely Random Forest (RF) and XGBoost, the latter proved more effective for both feature selection and model construction.
3. The application of SR methods during data preprocessing improved model precision. Specifically, data optimized using the GAN-based Real-ESRGAN method and the Transformer-based SwinIR method yielded better results than those processed by CNN-based methods such as SRCNN and EDSR.

The results of this research offer new insights into efficient UAV crop monitoring. By leveraging the enhancement capabilities of SR technology on UAV RGB images, UAVs can potentially operate at higher altitudes while maintaining high model precision. Combined with machine learning techniques, this provides an efficient method for monitoring soybean growth and managing agricultural fields.

## Data Availability

The datasets presented in this study can be found in online repositories. The names of the repository/repositories and accession number(s) can be found in the article/**Supplementary Material**.
