# Supplementary figures and images for: UAV multi-source data fusion with super-resolution for accurate soybean leaf area index estimation
Source: Front Plant Sci. 2025 Nov 20;16:1700660. doi: 10.3389/fpls.2025.1700660 (PMC12675413; doi:10.3389/fpls.2025.1700660)

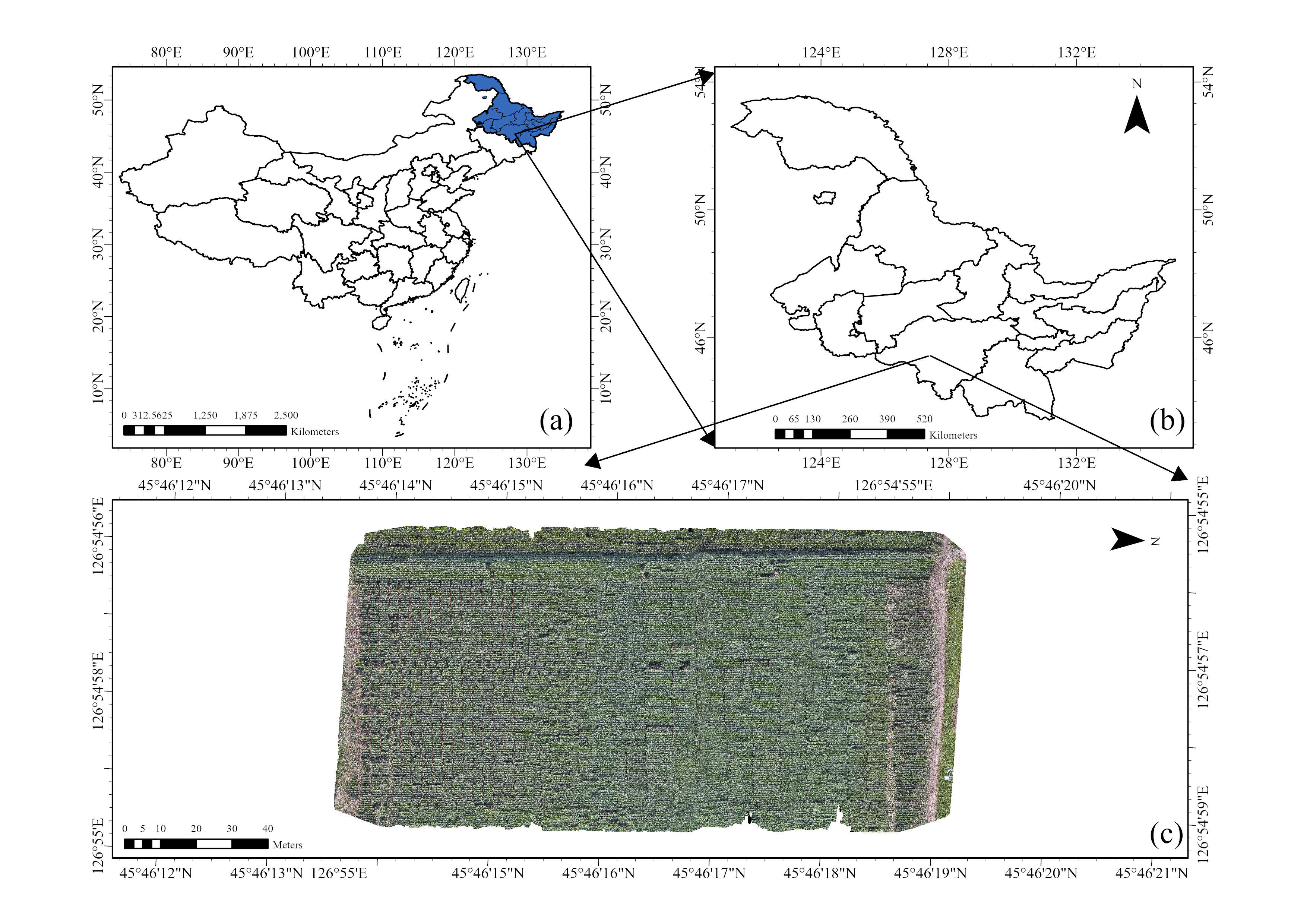

Supplement: Supplementary file 1 [file Image1.jpeg]

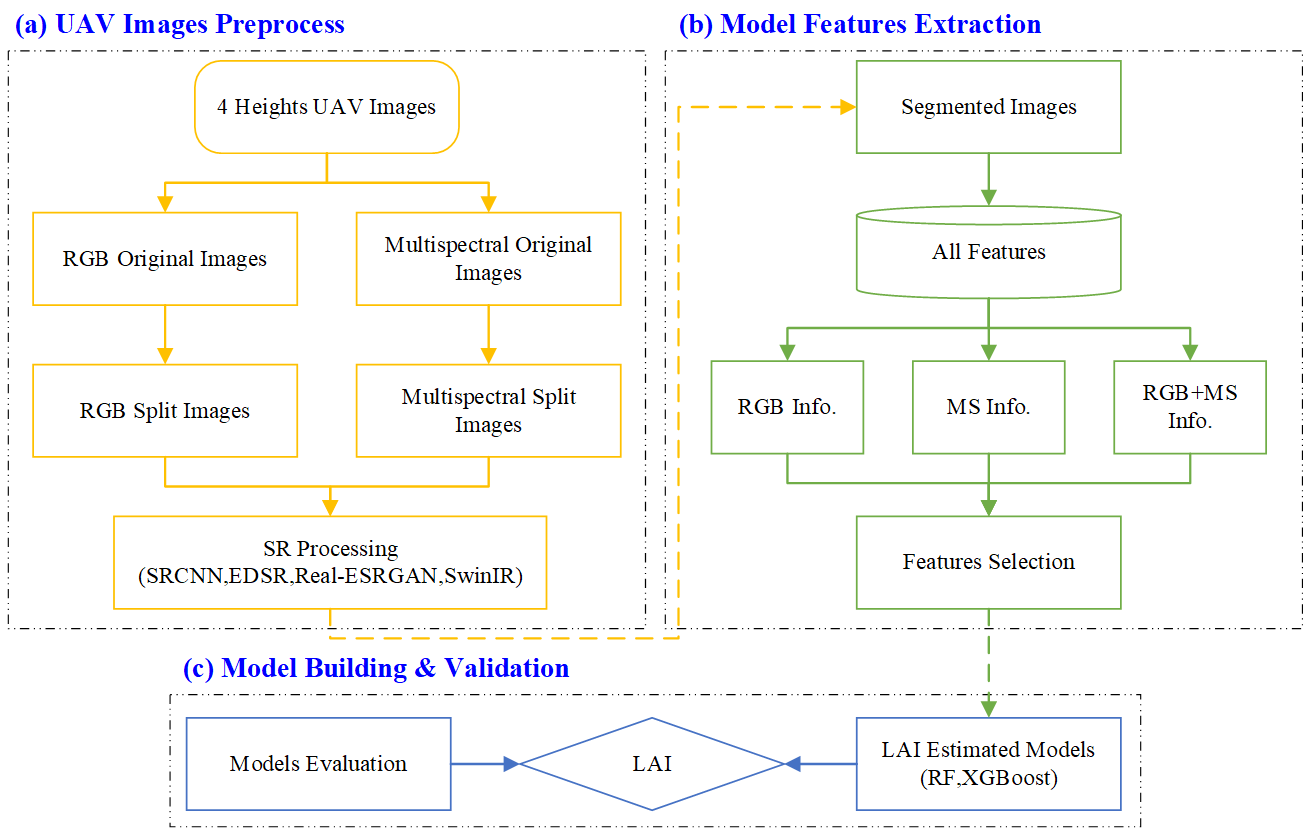

Supplement: Supplementary file 2 [file Image2.tif]

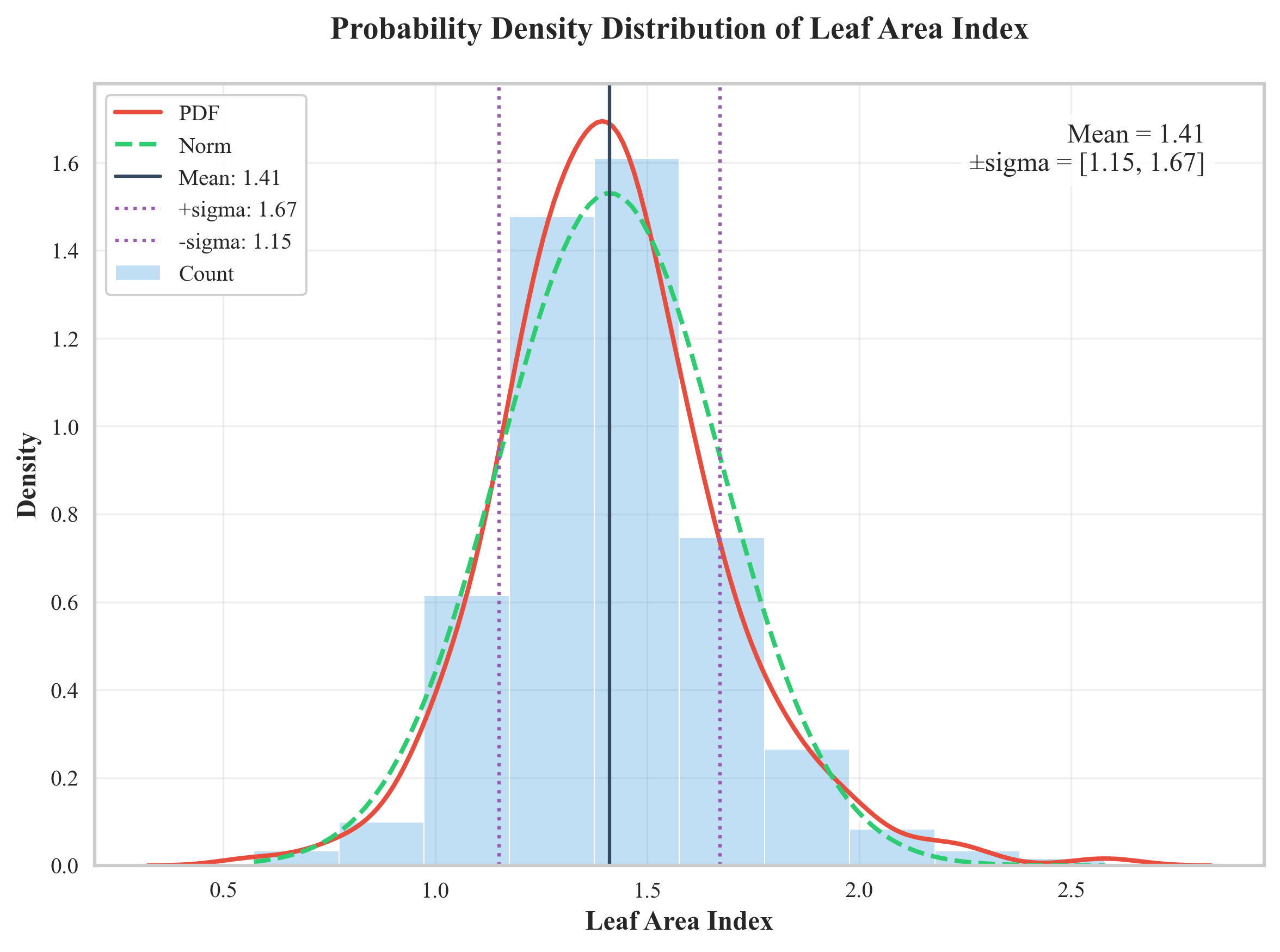

Supplement: Supplementary file 3 [file Image3.png]

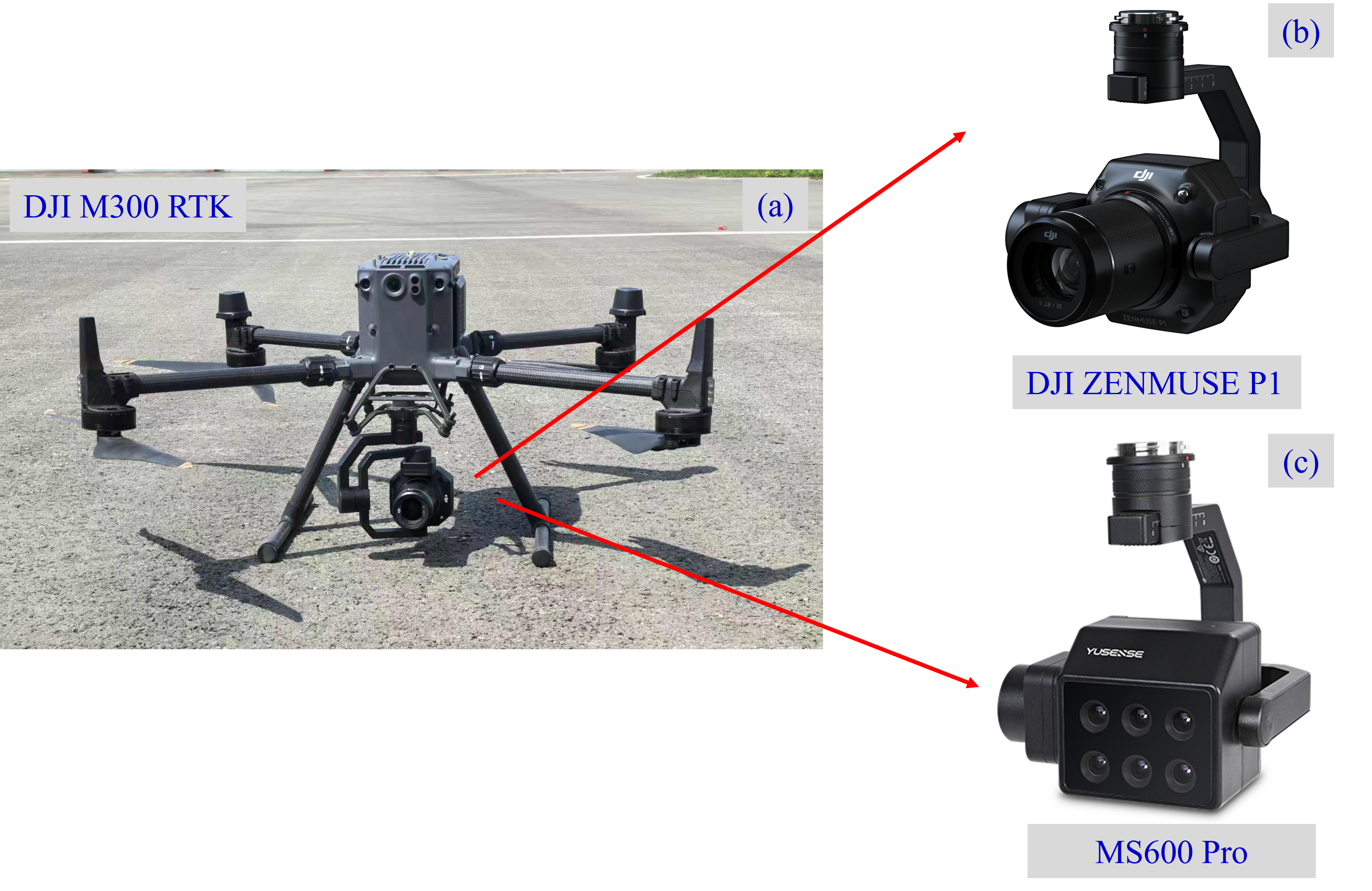

Supplement: Supplementary file 4 [file Image4.png]

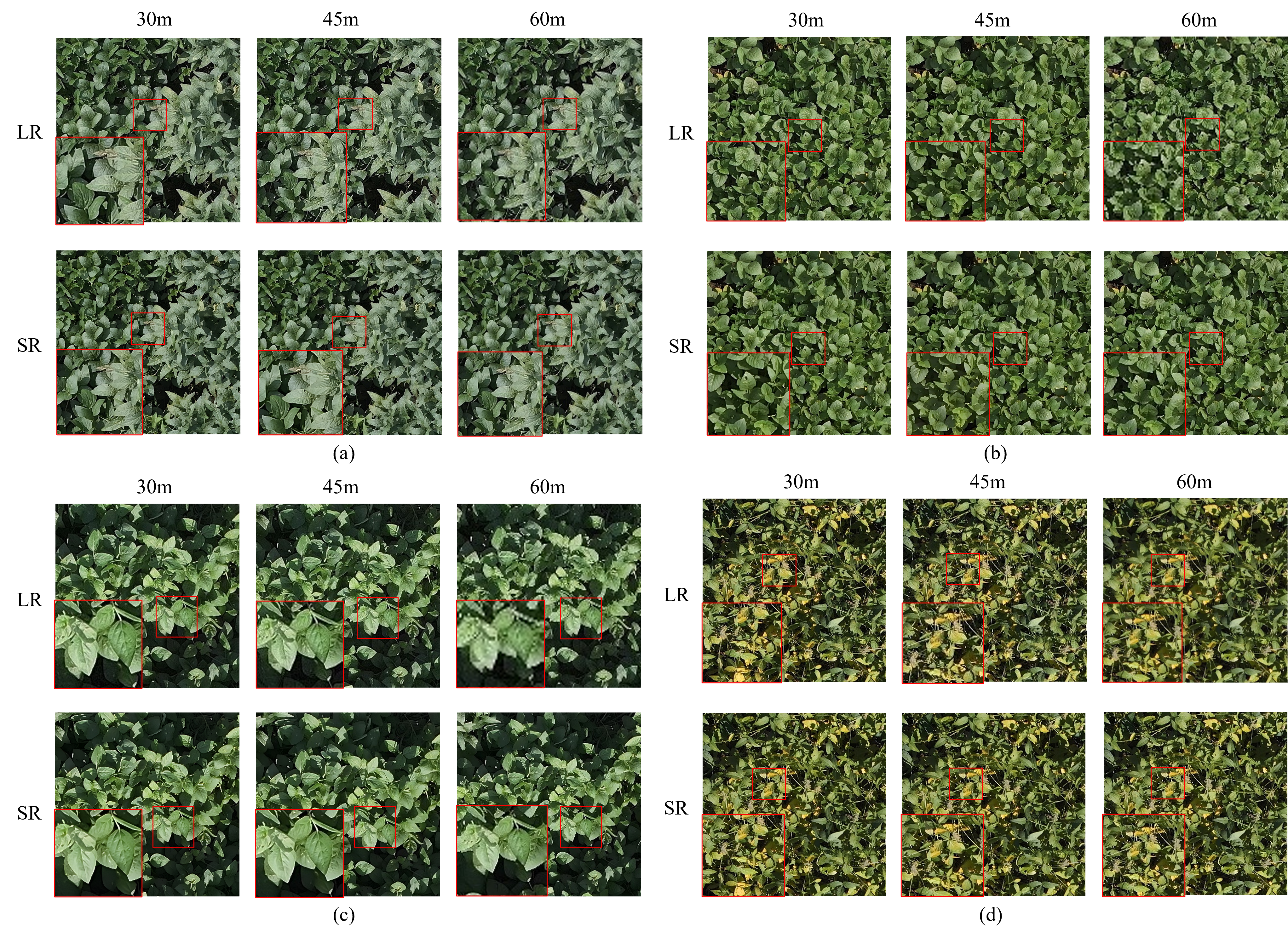

Supplement: Supplementary file 5 [file Image5.png]

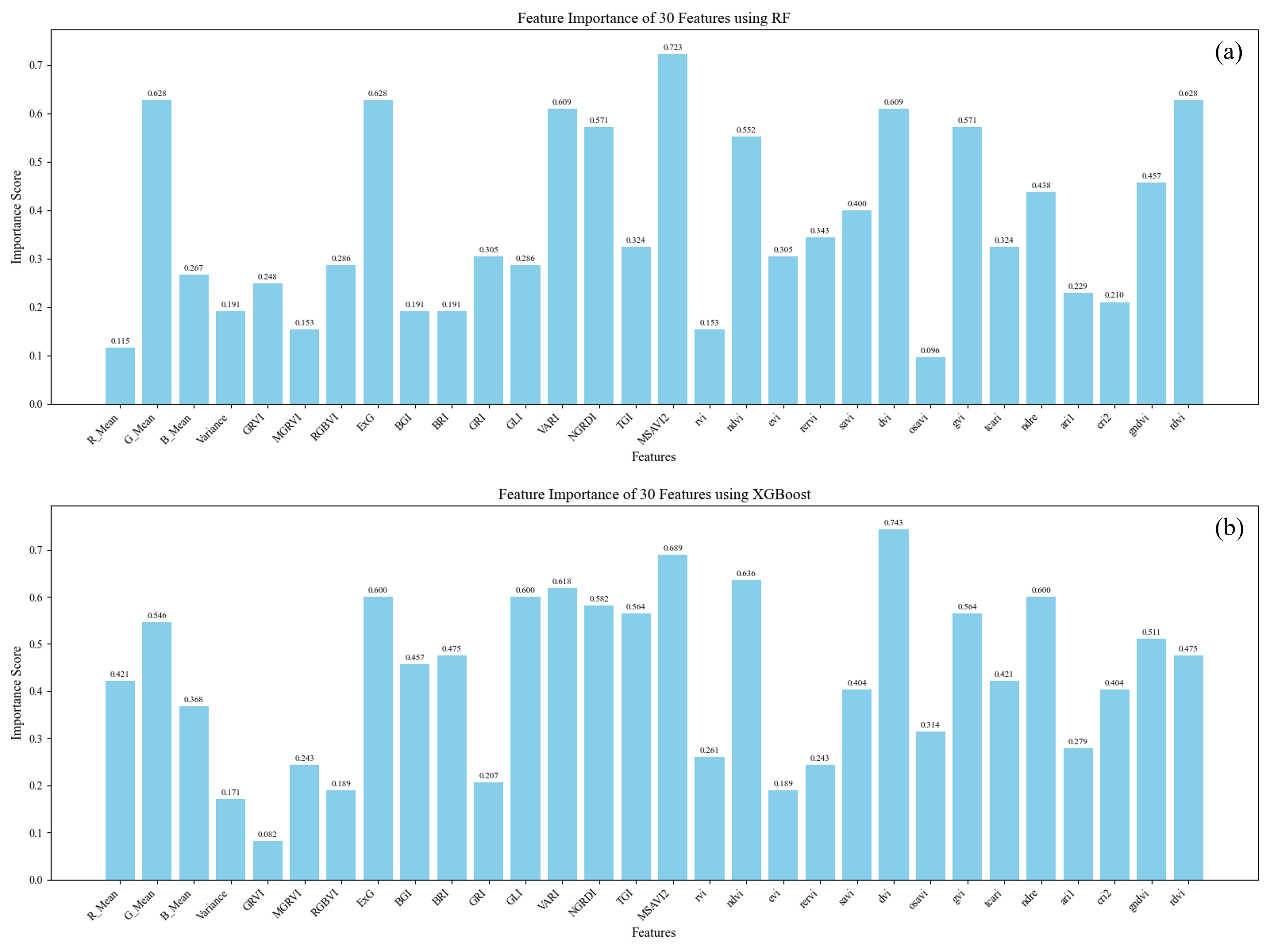

Supplement: Supplementary file 6 [file Image6.png]

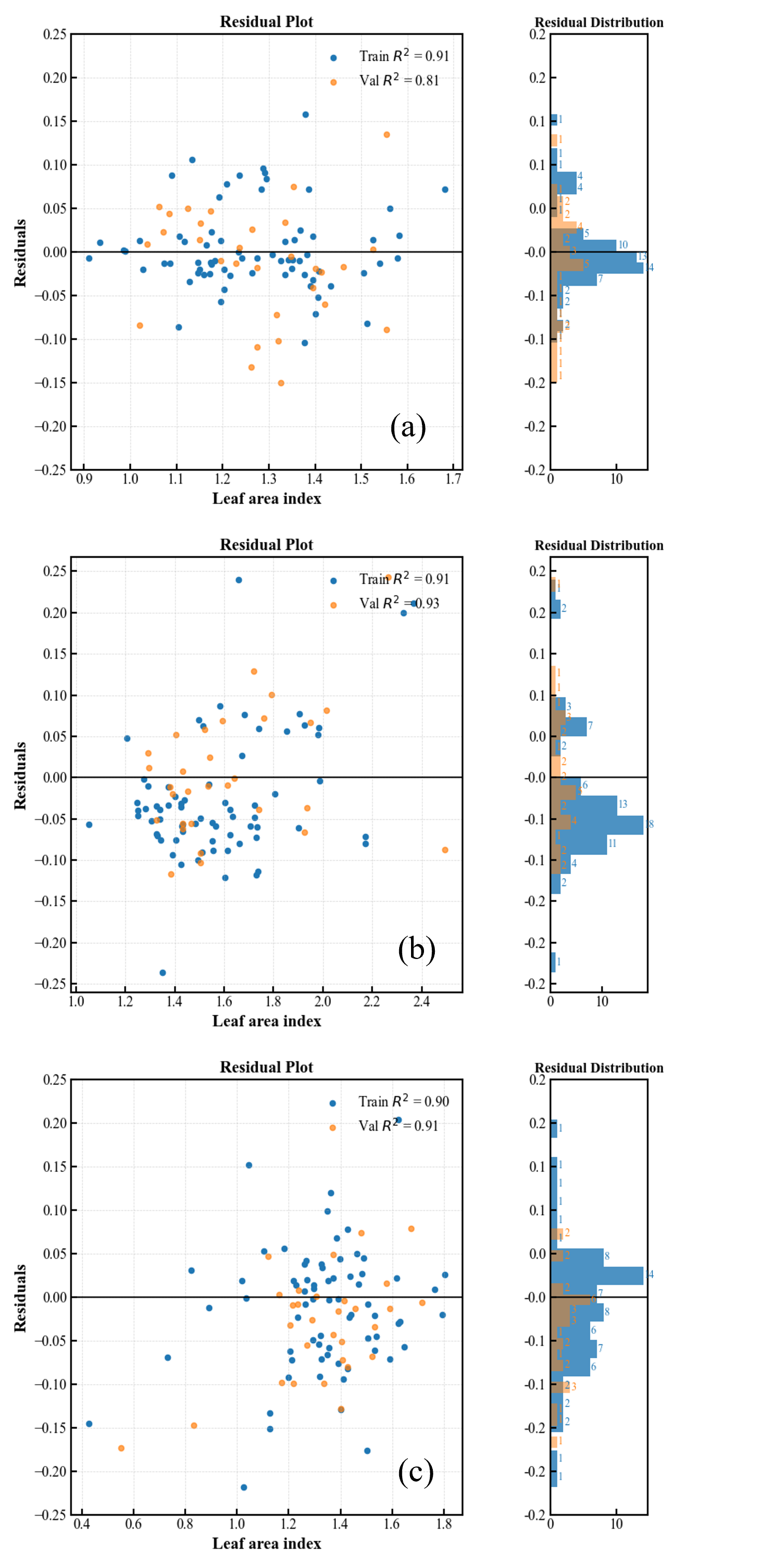

Supplement: Supplementary file 7 [file Image7.png]

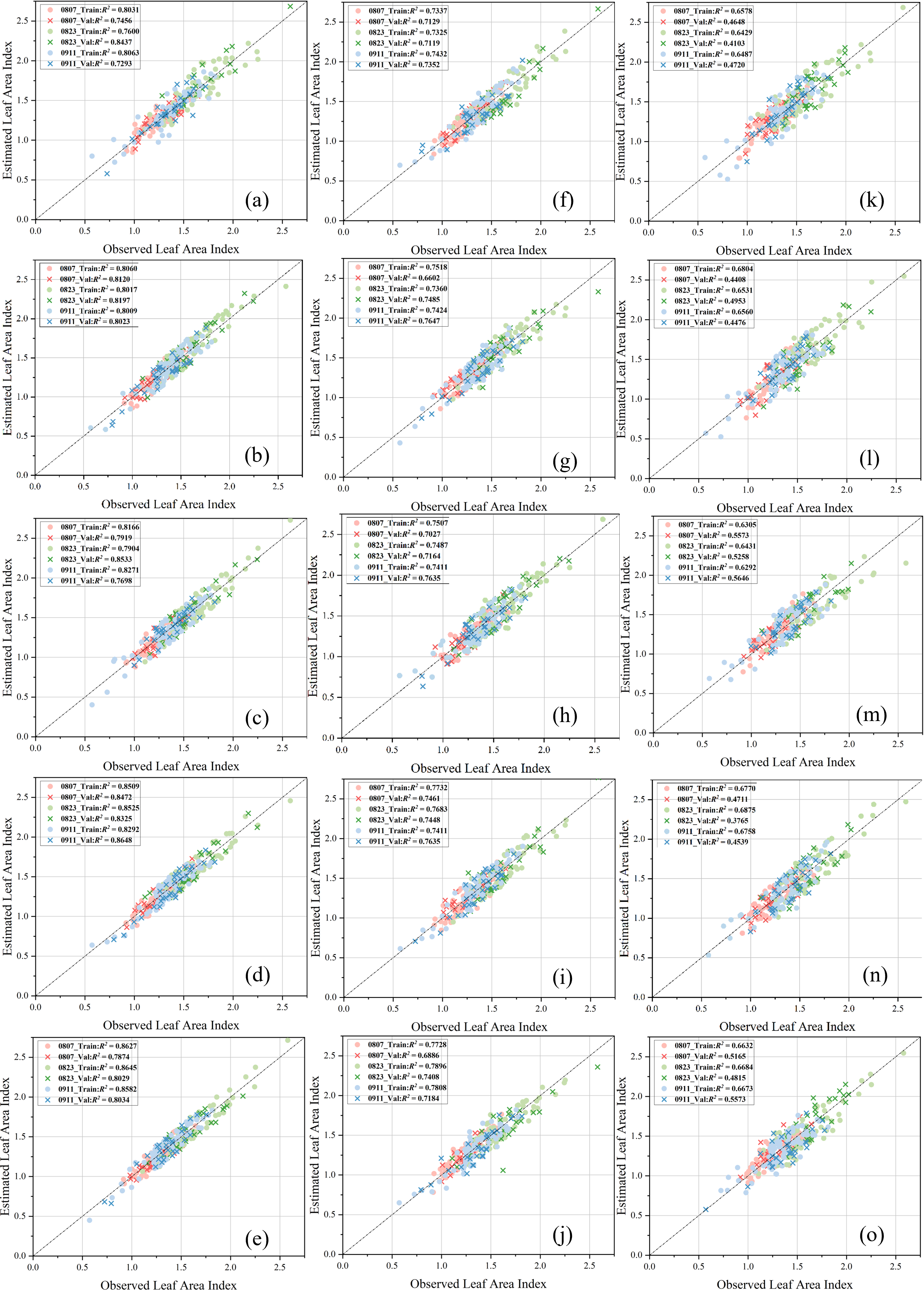

Supplement: Supplementary file 8 [file Image8.png]

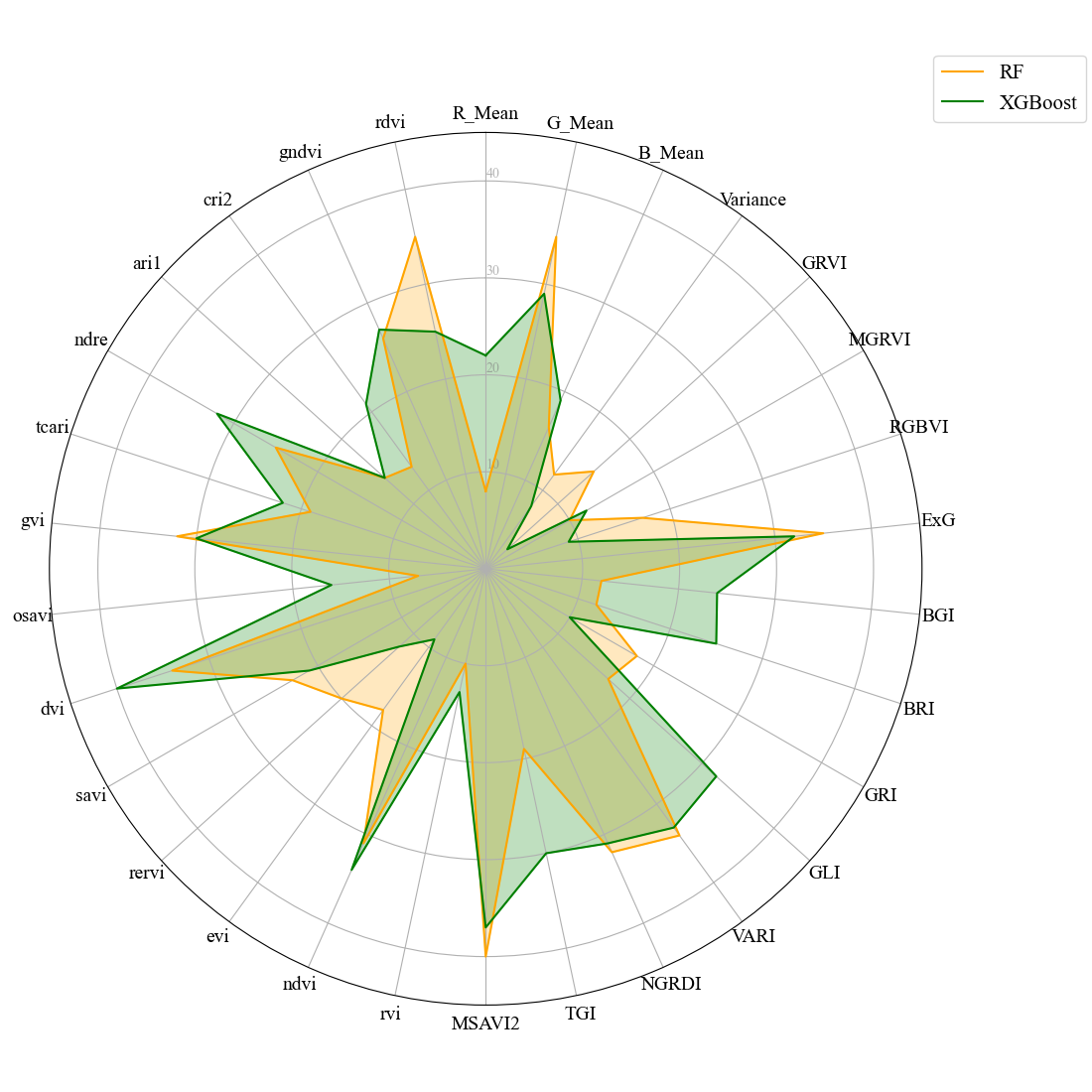

Supplement: Supplementary file 9 [file Image9.png]

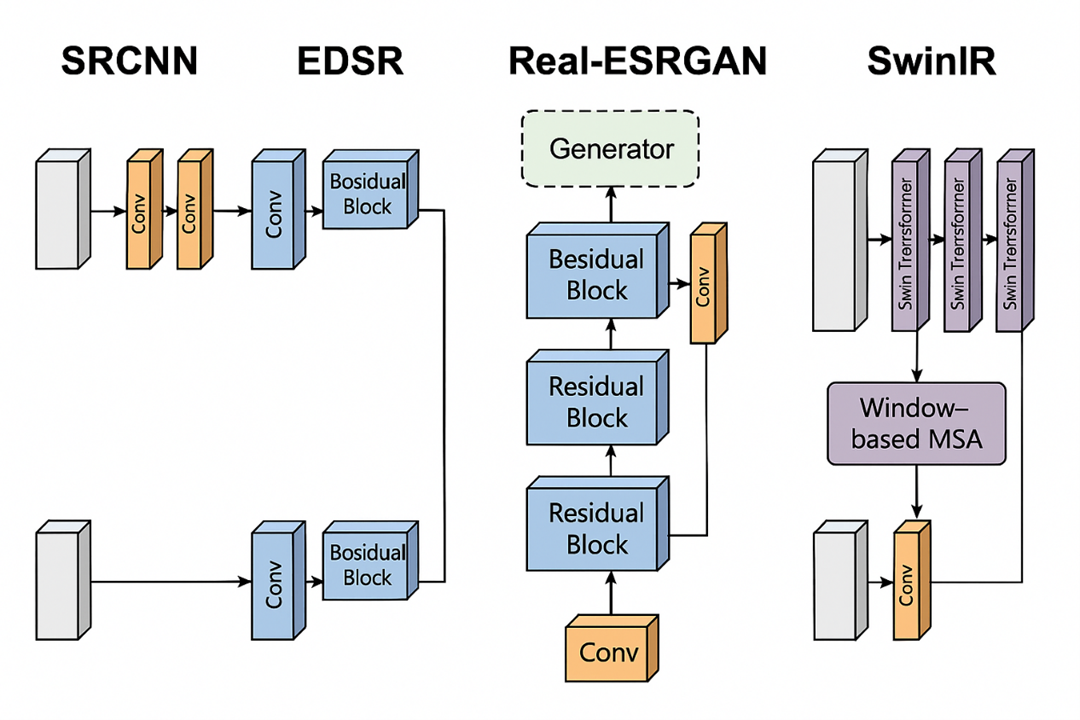

Supplement: Supplementary file 10 [file Image10.png]

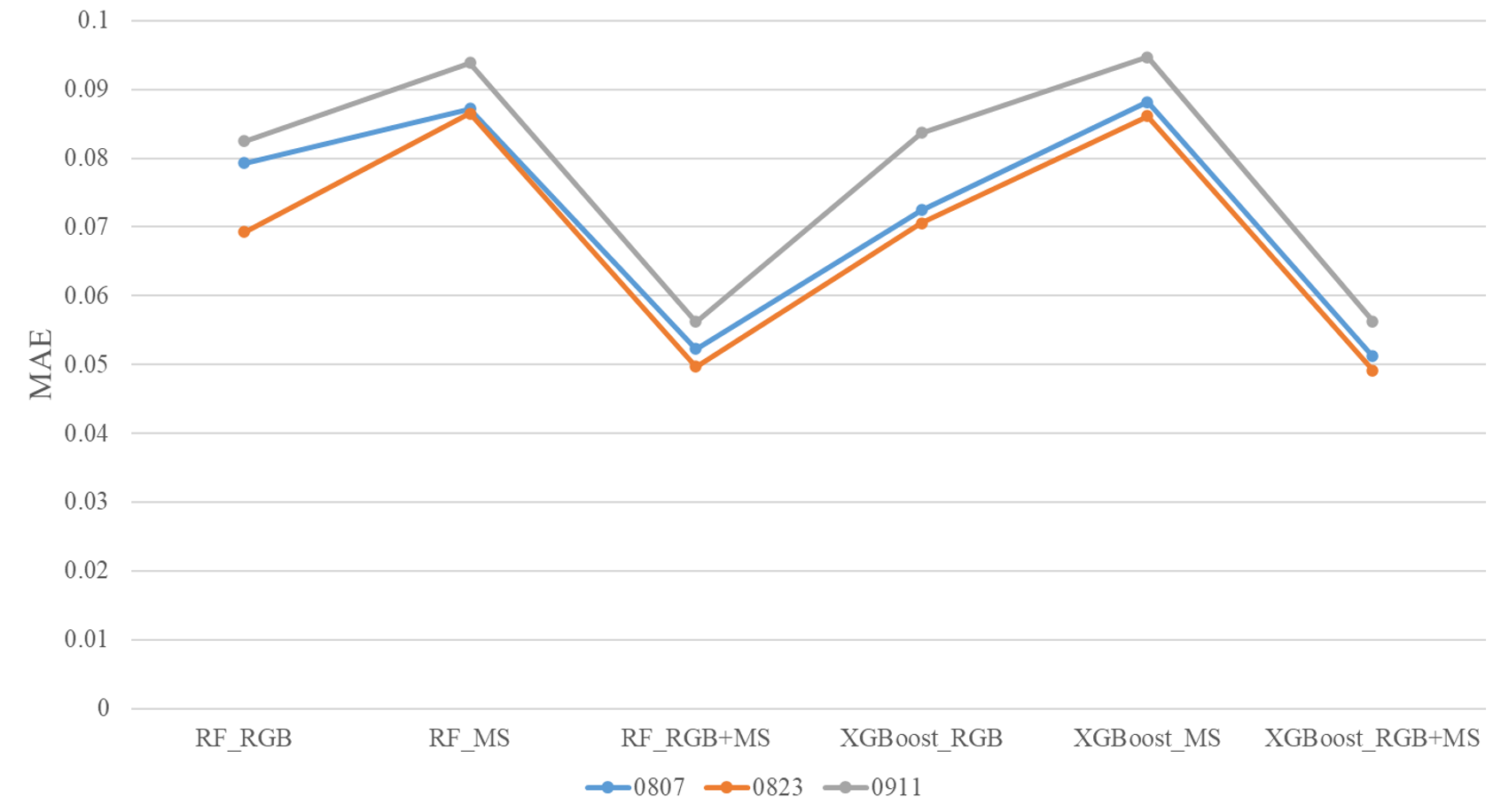

Supplement: Supplementary file 11 [file Image11.png]
