# Supplementary material for: UAV multi-source data fusion with super-resolution for accurate soybean leaf area index estimation
Source: Front Plant Sci. 2025 Nov 20;16:1700660. doi: 10.3389/fpls.2025.1700660 (PMC12675413; doi:10.3389/fpls.2025.1700660)
Supplement: Supplementary file 12 [file Table1.docx]

**Appendix**

Table A1 Modeling features derived from UAV RGB and multispectral images included in this study and their definitions.

| Features | Definition | References |
| --- | --- | --- |
| R_Mean |  | Basic statistical measure |
| G_Mean |  | Basic statistical measure |
| B_Mean |  | Basic statistical measure |
| Intensity variance |  | Basic statistical measure |
| Green-Red Vegetation Index |  | (Tucker, 1979) |
| Modified Green-Red Vegetation Index |  | (Bendig et al., 2015) |
| Red-Green-Blue Vegetation Index |  | (Louhaichi et al., 2001) |
| Excess Green Index |  | (Woebbecke et al., 1995) |
| Blue-Green Index |  | Basic statistical measure |
| Blue-Red Index |  | Basic statistical measure |
| Green-Red Index |  | (Tucker, 1979) |
| Triangular Greenness Index |  | (Hunt et al., 2013) |
| Green Leaf Index |  | (Louhaichi et al., 2001) |
| Visible Atmospherically Resistant Index |  | (Gitelson et al., 1996) |
| Normalized Green-Red Difference Index |  | (Tucker, 1979) |
| Modified Soil-Adjusted Vegetation Index 2 |  | (Qi et al., 1994) |
| Ratio Vegetation Index |  | (Pearson & Miller, 1972) |
| Normalized Difference Vegetation Index |  | (Rouse et al., 1974) |
| Enhanced Vegetation Index |  | (Huete et al., 2002) |
| Red Edge-Red Vegetation Index |  | Basic statistical measure |
| Soil-Adjusted Vegetation Index |  | (Huete, 1988) |
| Difference Vegetation Index |  | (Richardson & Wiegand, 1977) |
| Optimized Soil-Adjusted Vegetation Index |  | (Rondeaux et al., 1996) |
| Green Vegetation Index |  | Basic statistical measure |
| Transformed Chlorophyll Absorption Reflectance Index |  | (Haboudane et al., 2002) |
| Normalized Difference Red Edge Index |  | (Gitelson & Merzlyak, 1994) |
| Anthocyanin Reflectance Index 1 |  | (Gitelson et al., 2001) |
| Carotenoid Reflectance Index 2 |  | (Gitelson et al., 2001) |
| Green Normalized Difference Vegetation Index |  | (Gitelson et al., 1996) |
| Renormalized Difference Vegetation Index |  | (Roujean & Breon, 1995) |

**Reference**

Bendig, J., Yu, K., Aasen, H., et al. (2015). Combining UAV-based plant height from crop surface models, visible, and near infrared vegetation indices for biomass monitoring in barley. International Journal of Applied Earth Observation and Geoinformation, 39, 79–87.

Gitelson, A. A., & Merzlyak, M. N. (1994). Quantitative estimation of chlorophyll-a using reflectance spectra. Journal of Photochemistry and Photobiology B: Biology, 22(3), 247–252.

Gitelson, A. A., Kaufman, Y. J., & Merzlyak, M. N. (1996). Use of a green channel in remote sensing of global vegetation. Remote Sensing of Environment, 58(3), 289–298.

Gitelson, A. A., Keydan, G. P., & Merzlyak, M. N. (2001). Remote estimation of canopy chlorophyll content in crops. Geophysical Research Letters, 28(5), 779–782.

Haboudane, D., Miller, J. R., Tremblay, N., et al. (2002). Integrated narrow-band vegetation indices for prediction of crop chlorophyll content. Remote Sensing of Environment, 81(2-3), 416–426.

Hunt, E. R., Doraiswamy, P. C., McMurtrey, J. E., et al. (2013). A visible band index for remote sensing leaf chlorophyll content. International Journal of Applied Earth Observation and Geoinformation, 21, 103–112.

Huete, A. R. (1988). A soil-adjusted vegetation index (SAVI). Remote Sensing of Environment, 25(3), 295–309.

Huete, A., Didan, K., Miura, T., et al. (2002). Overview of the radiometric and biophysical performance of the MODIS vegetation indices. Remote Sensing of Environment, 83(1-2), 195–213.

Louhaichi, M., Borman, M. M., & Johnson, D. E. (2001). Spatially located platform and aerial photography for documentation of grazing impacts. Geocarto International, 16(1), 65–70.

Pearson, R. L., & Miller, L. D. (1972). Remote mapping of standing crop biomass. Proceedings of the Eighth International Symposium on Remote Sensing of Environment, 1357–1381.

Qi, J., Chehbouni, A., Huete, A. R., et al. (1994). A modified soil adjusted vegetation index. Remote Sensing of Environment, 48(2), 119–126.

Richardson, A. J., & Wiegand, C. L. (1977). Distinguishing vegetation from soil background information. Photogrammetric Engineering and Remote Sensing, 43(12), 1541–1552.

Rondeaux, G., Steven, M., & Baret, F. (1996). Optimization of soil-adjusted vegetation indices. Remote Sensing of Environment, 55(2), 95–107.

Rouse, J. W., Haas, R. H., Schell, J. A., & Deering, D. W. (1974). Monitoring vegetation systems in the Great Plains with ERTS. NASA Special Publication, 351, 309.

Roujean, J. L., & Breon, F. M. (1995). Estimating PAR absorbed by vegetation. Remote Sensing of Environment, 51(3), 375–384.

Tucker, C. J. (1979). Red and photographic infrared linear combinations for monitoring vegetation. Remote Sensing of Environment, 8(2), 127–150.

Woebbecke, D. M., Meyer, G. E., Von Bargen, K., & Mortensen, D. A. (1995). Color indices for weed identification. Transactions of the ASAE, 38(1), 259–269.
