# Supplementary material for: UAV multi-source data fusion with super-resolution for accurate soybean leaf area index estimation
Source: Front Plant Sci. 2025 Nov 20;16:1700660. doi: 10.3389/fpls.2025.1700660 (PMC12675413; doi:10.3389/fpls.2025.1700660)
Supplement: Supplementary file 13 [file Table2.docx]

**Appendix**

Table A2 List of the 100 soybean varieties used for model validation.

| **No.** | **Variety Name** |
| --- | --- |
| 1 | Longken 303 |
| 2 | Shendou No. 5 |
| 3 | G.maxN258/2006 |
| 4 | Kenjiandou No. 7 |
| 5 | Yushi 15 |
| 6 | Jifeng No. 1 |
| 7 | Kenjiandou 31 (Jiusan 95-88) |
| 8 | Changnong 21 |
| 9 | Tiefeng 30 |
| 10 | Kendou 63 |
| 11 | Xingdou No. 8 |
| 12 | Hefeng 17 |
| 13 | Ha 88-2499-Yellow |
| 14 | Dongnong 54 |
| 15 | Jinong 18 |
| 16 | Jiyu 95 |
| 17 | Jiunong 31 |
| 18 | Kenfeng 11 |
| 19 | Jiannong No. 1 |
| 20 | Jiyu 507 |
| 21 | Jiyu 202 |
| 22 | Ltocista |
| 23 | OAC Scorpio |
| 24 | G.max-25 |
| 25 | C∏1225 |
| 26 | MANDARIN HYBRID |
| 27 | CA37 |
| 28 | Amsoy 71 |
| 29 | Yyukimusume |
| 30 | C∏1422 |
| 31 | C∏1354 |
| 32 | Dongnong 48 |
| 33 | Jinong 45 |
| 34 | Kendou 39 |
| 35 | Dongsheng 22 |
| 36 | Heinong 82 |
| 37 | Kendou 61 |
| 38 | Mudou 13 |
| 39 | Qundou SLQ8 |
| 40 | Tiefeng 35 (Tie 95068-5) |
| 41 | Josefine |
| 42 | WDD01128 |
| 43 | Henong 123 |
| 44 | Kasota |
| 45 | Jiyu 87 |
| 46 | Canada No. 3 |
| 47 | Heinong 93 |
| 48 | Jinshan No. 3 |
| 49 | Anoka |
| 50 | Jinong 32 |
| 51 | C∏719 |
| 52 | Liaodou 21 |
| 53 | G.max-15 |
| 54 | WDD01107 |
| 55 | Tiedou 99 |
| 56 | WDD01157 |
| 57 | Jidadou 19 |
| 58 | VEGA |
| 59 | Jiyu 310 |
| 60 | Longken 304 |
| 61 | WDD01079 |
| 62 | Longken 302 |
| 63 | Changnong No. 9 |
| 64 | Jiyu 232 |
| 65 | PSB544 |
| 66 | Gnome |
| 67 | Heinong 46 (Ha 94-318) |
| 68 | Jinong 26 |
| 69 | SQWH |
| 70 | Canada 17 |
| 71 | Jiadou 33 |
| 72 | Jiyu 260 |
| 73 | OT93-28 |
| 74 | Henong 91 |
| 75 | Jilin 16 |
| 76 | WDD01125 |
| 77 | Heihe 18 |
| 78 | Longpin 96-303 |
| 79 | Jiyu 209 |
| 80 | MECHTA |
| 81 | Jiyu 209 |
| 82 | Liaodou 66 |
| 83 | Blackhawk |
| 84 | Liaodou 58 |
| 85 | Longdou No. 6 |
| 86 | OAC Stratford |
| 87 | Beiyi 27 |
| 88 | Jinong 54 |
| 89 | Bainong No. 6 |
| 90 | Baofeng No. 6 |
| 91 | Beidou 23 |
| 92 | Jilidou No. 3 |
| 93 | Jinong 13 |
| 94 | Jiyu 46 |
| 95 | Jiunong 28 |
| 96 | Kejiao 88223-1 |
| 97 | Liaodou 20 (Liao 95273) |
| 98 | Hardin |
| 99 | CA18 |
| 100 | Barnes |
